# Shifting competing underlying causes of death among U.S. decedents with diabetes recorded as a non-underlying cause, 1999–2024

**DOI:** 10.3389/fendo.2026.1957392

**Published:** 2026-09-30

**Authors:** Yanhua Xiao, Qian Zhou, Xi Yan, Lihui Gan, Tong Chen, Kaiyi Zhang, Renqing Zeng, Dibin Guo, Xuezhi Hong

**Affiliations:** 1 Department of Dermatology, The First Affiliated Hospital of Gannan Medical University, Ganzhou, China; 2 The First Clinical Medical College, Gannan Medical University, Ganzhou, China; 3 Department of Rheumatology and Immunology, The First Affiliated Hospital of Gannan Medical University, Ganzhou, China; 4 Department of Emergency Medicine, The First Affiliated Hospital of Gannan Medical University, Ganzhou, Jiangxi, China

**Keywords:** cardiovascular disease, diabetes mellitus, mortality, multiple cause of death, type 1 diabetes, type 2 diabetes, underlying cause of death

## Abstract

**Objective:**

To examine U.S. trends and underlying-cause patterns among deaths with diabetes recorded as a non-underlying cause (DM-nonUCD).

**Methods:**

We used CDC WONDER Multiple Cause of Death data from 1999 to 2024. DM-nonUCD was defined as deaths with ICD-10 codes E10-E14 in multiple-cause fields, excluding deaths with diabetes as the underlying cause. Analyses were performed overall and for type 1 diabetes (E10) and type 2 diabetes (E11). Death counts, age-adjusted mortality rates (AAMRs), and Joinpoint trends were assessed.

**Results:**

DM-nonUCD occurred in 4,794,136 deaths and increased from 141,371 in 1999 to 246,426 in 2024. The leading underlying causes were circulatory diseases (2,294,799; 47.87%), malignant neoplasms (725,592; 15.13%), and respiratory diseases (449,837; 9.38%). Circulatory diseases remained dominant but declined from 60.51% to 44.48%, with decreasing AAMR (average annual percent change [AAPC], -1.01%; 95% CI, -1.21 to -0.88). Shares increased for malignant neoplasms (14.01% to 16.30%), nervous system diseases (2.67% to 7.56%), and mental and behavioral disorders (1.05% to 4.41%). Within circulatory causes, ischemic heart disease AAMR declined (AAPC, -2.04%; 95% CI, -2.23 to -1.92), whereas hypertensive disease AAMR increased (AAPC, 3.21%; 95% CI, 2.94 to 3.56). Subtype analyses showed similar cause-distribution shifts for E10- and E11-nonUCD, with declining circulatory shares and increasing nervous system and mental and behavioral shares.

**Conclusions:**

DM-nonUCD represents a substantial proportion of deaths in which diabetes was recorded on the death certificate. The underlying-cause profile is shifting from coronary-dominated mortality toward broader multimorbidity, supporting surveillance and health-system planning that consider competing causes beyond ischemic heart disease.

## Introduction

Diabetes mellitus (DM) is a major chronic disease and an important contributor to mortality worldwide (1, 2). Its mortality burden extends beyond deaths directly attributed to diabetes. Long-term metabolic injury affects multiple organ systems, and diabetes is associated with excess mortality from vascular disease and several nonvascular causes, including cancer, infection, renal disease, liver disease, and nervous system disorders (3, 4). Over recent decades, however, diabetes care has changed substantially. Improvements in glycemic management, cardiovascular risk reduction, kidney protection, and acute care may have prolonged survival for many people with diabetes (5, 6). As patients live longer and accumulate comorbidities, the conditions ultimately selected as the underlying cause of death may also change.

Death-certificate studies commonly assess diabetes-related mortality using either deaths in which diabetes is selected as the underlying cause of death (DM-UCD) or deaths in which diabetes is mentioned anywhere on the death certificate (DM-AM) (7). These two measures capture different aspects of mortality surveillance. DM-UCD is specific to deaths directly assigned to diabetes, whereas DM-AM is broader and includes both underlying-cause deaths and deaths in which diabetes was recorded as a contributing condition. Diabetes recorded as a non-underlying cause of death (DM-nonUCD) isolates the latter category and refers to deaths in which diabetes is mentioned on the death certificate but another condition is selected as the underlying cause. This mutually exclusive category allows the competing underlying causes of death to be examined among decedents for whom diabetes was recorded as part of the causal context.

Contemporary national trends in DM-nonUCD and the distribution of competing underlying causes remain insufficiently characterized, particularly as diabetes care and cardiovascular prevention have evolved over time (4, 8). These changes may influence which conditions are selected as underlying causes when diabetes is recorded as a contributing condition. At the same time, people with diabetes may remain vulnerable to long-term consequences of cumulative metabolic and vascular injury. Type 1 DM and type 2 DM differ in age at onset, disease duration, treatment patterns, metabolic characteristics, and complication profiles (8, 9). These differences may lead to different competing-cause mortality patterns. Using U.S. national multiple-cause-of-death data from 1999 through 2024, we examined deaths in which diabetes was recorded as a non-underlying cause of death. We evaluated temporal patterns overall and by diabetes subtype. We also characterized the competing underlying-cause distribution, with particular attention to circulatory disease subtypes. We aimed to determine whether the mortality profile of DM-nonUCD remains predominantly cardiovascular or has shifted toward a broader multimorbidity-related pattern.

## Research design and methods

### Study design and data source

We performed a population-based repeated cross-sectional study using U.S. death certificate data from 1999 to 2024. Data for 1999–2020 were obtained from the CDC WONDER Multiple Cause of Death, 1999–2020 database, and data for 2021–2024 from the Multiple Cause of Death, 2018–2024 database. For the overlapping years 2018–2020, the former database was used. Query settings are provided in **Supplementary Table 1**. The study period was limited to years coded using ICD-10.

Each death certificate includes one underlying cause of death (UCD) and multiple causes of death (MCOD). The UCD is the condition that initiated the sequence of events leading directly to death, whereas MCOD includes the UCD and other conditions recorded by the certifier. CDC WONDER provides death counts, population denominators, age-adjusted mortality rates (AAMRs), and standard errors. All data were publicly available, deidentified, and aggregated; therefore, institutional review board approval and informed consent were not required. The study was reported in accordance with the STROBE statement and the RECORD extension.

### Study population

The source population included all U.S. resident decedents recorded in the Multiple Cause of Death files during 1999–2024. The main analytic population included deaths in which any diabetes code (ICD-10 E10-E14) appeared in the MCOD fields but no diabetes code was selected as the UCD. These deaths were defined as DM-nonUCD. Deaths with diabetes as the UCD were excluded.

The primary analysis used all diabetes codes combined (E10-E14). Subtype analyses were conducted for type 1 diabetes (E10) and type 2 diabetes (E11). For these analyses, deaths were included in the E10- or E11-specific analysis when the corresponding code appeared in the MCOD fields, after excluding all deaths with any diabetes code as the UCD. Because E10- and E11-specific analyses were mention-based, the subtype categories were not assumed to be mutually exclusive, and subtype-specific estimates were not summed.

### Underlying-cause classification

Among DM-nonUCD deaths, the UCD was grouped into major ICD-10 cause categories, including infectious and parasitic diseases, malignant neoplasms, mental and behavioral disorders, diseases of the nervous system, circulatory diseases, respiratory diseases, digestive diseases, genitourinary diseases, external causes, and COVID-19. Circulatory deaths were further classified as hypertensive disease, ischemic heart disease, peripheral vascular disease, heart failure, and cerebrovascular disease. The ICD-10 code lists are shown in **Supplementary Table 2**.

### Mortality measures

We obtained annual death counts, AAMRs, and 95% confidence intervals from CDC WONDER. Rates were expressed per 100,000 U.S. residents and should be interpreted as population-level mortality rates, not as mortality risks among people living with diabetes. AAMRs were standardized to the 2000 U.S. standard population. We also calculated the annual proportion of all U.S. resident deaths represented by DM-nonUCD by dividing the annual number of DM-nonUCD deaths by the total number of deaths in the corresponding year.

Cause-specific proportions were calculated by dividing the number of DM-nonUCD deaths in a given UCD category by the total number of DM-nonUCD deaths in the corresponding year or analytic group. Cause distributions were also examined separately for deaths at ages <65 and ≥65 years. Within each age group, cause-specific proportions were calculated using all DM-nonUCD deaths in that group and study period as the denominator. Deaths with age not stated were excluded. Results were summarized for 1999–2004, 2005–2009, 2010–2014, 2015–2019, and 2020–2024. The same analysis was performed for E10-nonUCD and E11-nonUCD deaths. For circulatory subtypes, proportions were calculated relative to all DM-nonUCD deaths and, where appropriate, relative to deaths with circulatory disease as the UCD.

Cells suppressed by CDC WONDER because of small counts or confidentiality rules were treated as unavailable and were not used for rate estimation or trend modelling. National annual estimates were used for the primary trend analyses.

### Trend analysis

Temporal trends in annual AAMRs were analyzed using Joinpoint Regression Program version 5.4.0 (10). Log-linear models were fitted with calendar year as the independent variable and the natural logarithm of the AAMR as the dependent variable. Annual standard errors exported from CDC WONDER were used to account for heteroscedasticity, and errors were assumed to be uncorrelated.

Candidate models were fitted using grid search. For the 1999–2024 series, the default software settings allowed 0 to 4 joinpoints, with at least two observations required before the first joinpoint, after the last joinpoint, and between adjacent joinpoints. We retained these settings for the primary analysis to avoid segments based on a single observation. The final model was selected using the weighted Bayesian Information Criterion (WBIC), which accounts for both model fit and complexity (11). In a sensitivity analysis, the models were refitted using a maximum of two joinpoints, with at least three observations required before the first joinpoint, after the last joinpoint, and between adjacent joinpoints.

Annual percent change (APC) and average annual percent change (AAPC), with 95% confidence intervals, were estimated from the final selected model. The AAPC was calculated as a weighted average of the segment-specific APCs over the full study period. APC and AAPC estimates were considered statistically significant when their 95% confidence intervals did not include 0.

Joinpoint analyses were performed for overall DM-nonUCD deaths, diabetes subtypes, major UCD categories, and selected circulatory disease subtypes, both overall and by sex. Trend results were interpreted together with annual death counts and cause-specific proportions because rates, counts, and proportional composition may change in different directions.

As a sensitivity analysis, we repeated selected Joinpoint trend analyses after restricting the study period to 1999–2019 to assess whether the main cause-specific patterns were already present before the COVID-19 pandemic.

### Data access and reproducibility

Data were accessed through aggregate queries in CDC WONDER. No individual-level records were downloaded, and no linkage to external datasets was performed. The query definitions and ICD-10 code lists used for case identification and underlying-cause classification are provided in the **Supplementary Materials**. Extracted analytic tables and Joinpoint input files are available from the corresponding author upon reasonable request.

## Results

### Overall burden and diabetes subtype composition

From 1999 through 2024, diabetes was recorded as a non-underlying cause of death in 4,794,136 U.S. deaths (**Supplementary Table 3**). Annual DM-nonUCD deaths increased from 141,371 in 1999 to 246,426 in 2024, while their proportion among all U.S. resident deaths increased from 5.91% to 8.02% (**Supplementary Tables 3**, **4**). The pattern of recorded diabetes subtype mentions changed substantially over time. Deaths with E10 recorded as a non-underlying cause decreased from 12,438 to 3,419, and their share of all DM-nonUCD deaths declined from 8.80% to 1.39% (**Supplementary Figure 1**; **Supplementary Table 5**). In contrast, deaths with E11 recorded as a non-underlying cause increased from 27,915 to 110,757, and their share rose from 19.75% to 44.94% (**Supplementary Figure 1**; **Supplementary Table 6**).

### Underlying-cause distribution and AAMR trends in DM-nonUCD mortality

Circulatory diseases were the leading underlying cause among DM-nonUCD deaths, accounting for 2,294,799 deaths during 1999–2024, or 47.87% of the total (**Table 1**; **Supplementary Table 3**),. Malignant neoplasms were the second largest category, followed by respiratory diseases, diseases of the nervous system, mental and behavioral disorders, and digestive diseases (**Table 1**; **Supplementary Table 3**).

**Table 1:** Period-specific underlying-cause distribution of DM-nonUCD deaths, United States, 1999–2024.

Condition	1999–2004	2005–2009	2010–2014	2015–2019	2020–2024	Total
A00–B99	23597 (2.66%)	23105 (2.88%)	23545 (2.81%)	23187 (2.51%)	28569 (2.13%)	122003 (2.54%)
C00–C97	129711 (14.60%)	125728 (15.67%)	134822 (16.10%)	144953 (15.66%)	190378 (14.20%)	725592 (15.13%)
D00-D48	4169 (0.47%)	4119 (0.51%)	4311 (0.51%)	4537 (0.49%)	5526 (0.41%)	22662 (0.47%)
D50-D89	3193 (0.36%)	3111 (0.39%)	3411 (0.41%)	3694 (0.40%)	4755 (0.35%)	18164 (0.38%)
E00–E09, E15–E88	12777 (1.44%)	13890 (1.73%)	16537 (1.98%)	21268 (2.30%)	33718 (2.51%)	98190 (2.05%)
F01–F99	12104 (1.36%)	27484 (3.43%)	45393 (5.42%)	45378 (4.90%)	55400 (4.13%)	185759 (3.87%)
G00–G98	29169 (3.28%)	36838 (4.59%)	45370 (5.42%)	60892 (6.58%)	87459 (6.52%)	259728 (5.42%)
I00–I99	509213 (57.32%)	404057 (50.36%)	398977 (47.65%)	435521 (47.07%)	547031 (40.80%)	2294799 (47.87%)
J00–J98	80222 (9.03%)	81199 (10.12%)	87058 (10.40%)	94713 (10.24%)	106645 (7.95%)	449837 (9.38%)
K00–K92	32074 (3.61%)	29630 (3.69%)	30448 (3.64%)	34854 (3.77%)	49374 (3.68%)	176380 (3.68%)
L00-L98	1580 (0.18%)	2358 (0.29%)	2573 (0.31%)	2975 (0.32%)	4371 (0.33%)	13857 (0.29%)
M00-M99	6290 (0.71%)	6042 (0.75%)	6087 (0.73%)	6527 (0.71%)	10010 (0.75%)	34956 (0.73%)
N00–N98	28208 (3.18%)	24804 (3.09%)	12586 (1.50%)	11393 (1.23%)	15669 (1.17%)	92660 (1.93%)
Q00-Q99	877 (0.10%)	762 (0.09%)	884 (0.11%)	1010 (0.11%)	1442 (0.11%)	4975 (0.10%)
U07.1	0 (0.00%)	0 (0.00%)	0 (0.00%)	0 (0.00%)	147171 (10.98%)	147171 (3.07%)
Others	15218 (1.71%)	19193 (2.39%)	25265 (3.02%)	34438 (3.72%)	53289 (3.97%)	147403 (3.07%)
NonUCD	888402	802320	837267	925340	1340807	4794136

“Others” was calculated as the corresponding nonUCD total minus the sum of deaths assigned to A00–B99, C00–C97, D00–D48, D50–D89, E00–E09 and E15–E88, F01–F99, G00–G98, I00–I99, J00–J98, K00–K92, L00–L98, M00–M99, N00–N98, Q00–Q99, and U07.1 as the underlying cause of death.

The underlying-cause profile shifted away from circulatory disease over time. The proportion of DM-nonUCD deaths attributed to circulatory diseases decreased from 60.51% in 1999 to 44.48% in 2024 (**Supplementary Table 3**). In contrast, the proportions attributed to malignant neoplasms, diseases of the nervous system, and mental and behavioral disorders increased from 14.01% to 16.30%, from 2.67% to 7.56%, and from 1.05% to 4.41%, respectively (**Supplementary Table 3**). COVID-19 emerged during the pandemic years, accounted for 19.80% of DM-nonUCD deaths in 2020 and 19.90% in 2021, and then declined to 1.30% in 2024 (**Supplementary Table 3**). Period-based analyses showed the same redistribution (**Table 1**). The circulatory share decreased from 57.32% in 1999–2004 to 40.80% in 2020–2024, whereas the shares of nervous system diseases and mental and behavioral disorders increased (**Table 1**; **Supplementary Table 3**). A similar decline in the circulatory share was observed for both E10 and E11 (**Table 2**; **Supplementary Table 7**).

**Table 2:** Period-specific underlying-cause distribution of E11-nonUCD deaths, United States, 1999–2024.

Condition	1999–2004	2005–2009	2010–2014	2015–2019	2020–2024	Total
A00–B99	4497 (2.24%)	5836 (2.67%)	6607 (2.73%)	8034 (2.36%)	11158 (1.95%)	36132 (2.30%)
C00–C97	34647 (17.27%)	39629 (18.11%)	44951 (18.54%)	62940 (18.51%)	94564 (16.55%)	276731 (17.59%)
D00-D48	1053 (0.52%)	1307 (0.60%)	1440 (0.59%)	1893 (0.56%)	2745 (0.48%)	8438 (0.54%)
D50-D89	705 (0.35%)	919 (0.42%)	1092 (0.45%)	1401 (0.41%)	2193 (0.38%)	6310 (0.40%)
E00–E09, E15–E88	3403 (1.70%)	4363 (1.99%)	5573 (2.30%)	8964 (2.64%)	16796 (2.94%)	39099 (2.49%)
F01–F99	3215 (1.60%)	8440 (3.86%)	14302 (5.90%)	17750 (5.22%)	25476 (4.46%)	69183 (4.40%)
G00–G98	8994 (4.48%)	13321 (6.09%)	17577 (7.25%)	29126 (8.57%)	48161 (8.43%)	117179 (7.45%)
I00–I99	107359 (53.51%)	101039 (46.17%)	104259 (43.01%)	144989 (42.65%)	214481 (37.55%)	672127 (42.73%)
J00–J98	19510 (9.73%)	24055 (10.99%)	27272 (11.25%)	37254 (10.96%)	48463 (8.48%)	156554 (9.95%)
K00–K92	7312 (3.64%)	8318 (3.80%)	9385 (3.87%)	13377 (3.93%)	21998 (3.85%)	60390 (3.84%)
L00-L98	284 (0.14%)	603 (0.28%)	697 (0.29%)	1073 (0.32%)	1712 (0.30%)	4369 (0.28%)
M00-M99	1441 (0.72%)	1646 (0.75%)	1887 (0.78%)	2552 (0.75%)	4508 (0.79%)	12034 (0.77%)
N00–N98	5743 (2.86%)	6438 (2.94%)	3727 (1.54%)	4278 (1.26%)	6555 (1.15%)	26741 (1.70%)
Q00-Q99	193 (0.10%)	220 (0.10%)	251 (0.10%)	368 (0.11%)	657 (0.12%)	1689 (0.11%)
U07.1	0 (0.00%)	0 (0.00%)	0 (0.00%)	0 (0.00%)	59410 (10.40%)	59410 (3.78%)
Others	2259 (1.13%)	2710 (1.24%)	3392 (1.40%)	5971 (1.76%)	12346 (2.16%)	26678 (1.70%)
NonUCD	200615	218844	242412	339970	571223	1573064

“Others” was calculated as the corresponding nonUCD total minus the sum of deaths assigned to A00–B99, C00–C97, D00–D48, D50–D89, E00–E09 and E15–E88, F01–F99, G00–G98, I00–I99, J00–J98, K00–K92, L00–L98, M00–M99, N00–N98, Q00–Q99, and U07.1 as the underlying cause of death.

The decline in the circulatory share was observed in both age groups. Among DM-nonUCD deaths at ages <65 years, the proportion attributed to circulatory diseases decreased from 56.54% in 1999–2004 to 42.31% in 2020–2024 (**Supplementary Table 8**). Among deaths at ages ≥65 years, it decreased from 57.51% to 40.39%. Changes in mental and behavioral disorders and diseases of the nervous system were more marked in the older group (**Supplementary Table 8**). At ages ≥65 years, their respective proportions increased from 1.48% to 4.77% and from 3.64% to 7.72%; at ages <65 years, they increased from 0.89% to 1.77% and from 1.86% to 2.10% (**Supplementary Table 8**). Similar patterns were observed in the E10-nonUCD and E11-nonUCD analyses (**Supplementary Tables 9**, **10**).

AAMR-based Joinpoint analyses showed that these proportional changes reflected divergent rate trends. Circulatory disease-related DM-nonUCD mortality decreased from 31.32 to 24.84 per 100,000 U.S. residents, corresponding to an AAPC of -1.01% (95% CI, -1.21 to -0.88), indicating an average annual decrease of approximately 1.0% over the study period (**Figure 1A**). In contrast, AAMRs increased for several non-cardiovascular causes. The largest relative increases were observed for mental and behavioral disorders (AAPC, 6.73%; 95% CI, 5.88 to 8.40) and diseases of the nervous system (AAPC, 4.55%; 95% CI, 4.27 to 4.93) (**Figure 1B** and C). Malignant neoplasm-related mortality also increased modestly (AAPC, 0.76%; 95% CI, 0.61 to 0.90) (**Figure 1D**). Respiratory and digestive disease-related mortality showed smaller increases (**Figures 1E, F**).

**Figure 1:**
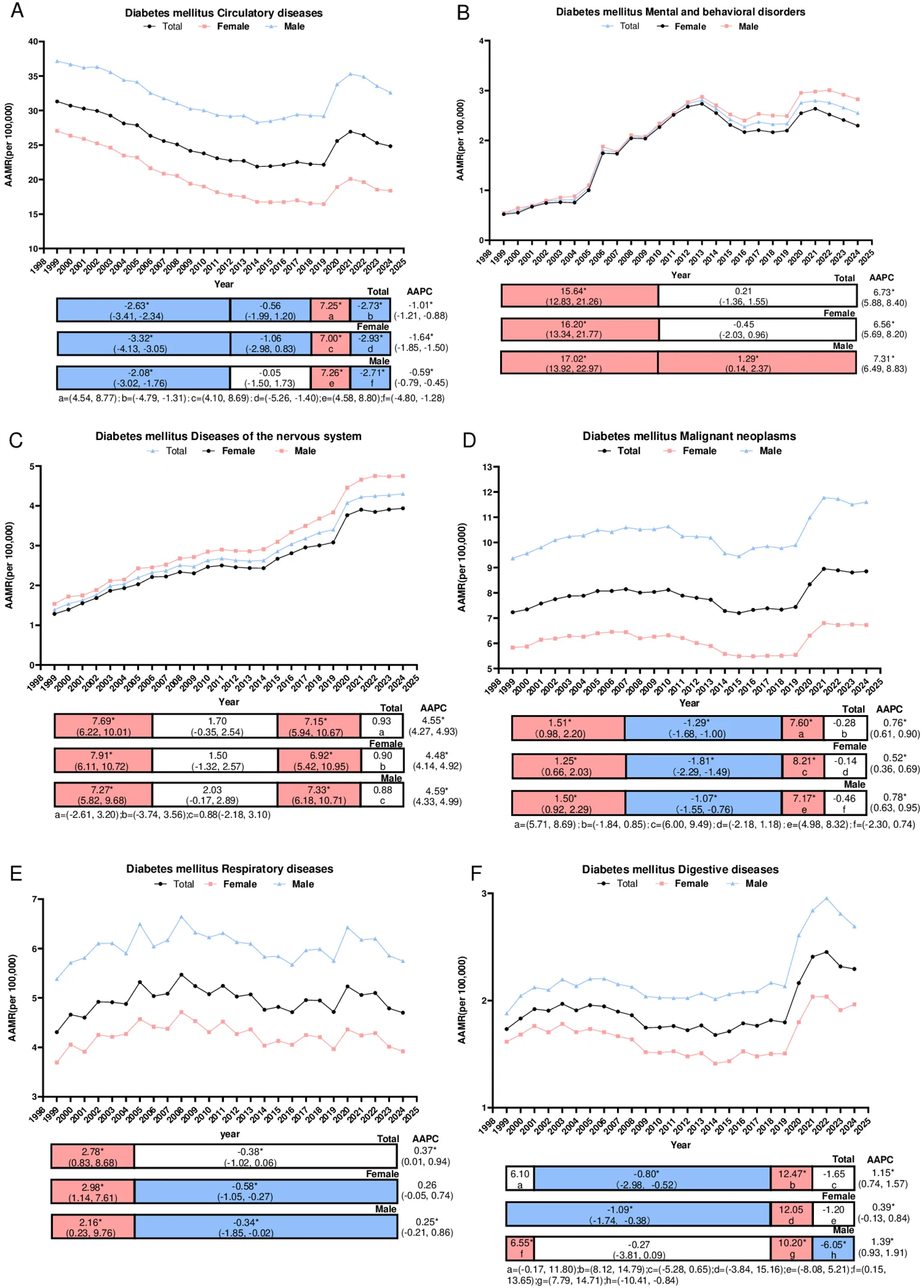
Age-adjusted mortality trends for selected underlying causes among DM-nonUCD deaths, United States, 1999–2024. Annual age-adjusted mortality rates (AAMR) for underlying causes assigned to **(A)** circulatory diseases, **(B)** mental and behavioral disorders, **(C)** diseases of the nervous system, **(D)** malignant neoplasms, **(E)** respiratory diseases, and **(F)** digestive diseases. Total estimates are shown together with sex-specific estimates for reference. Joinpoint segment bars below each plot indicate annual percentage changes (APC) for each fitted segment. Average annual percentage changes (AAPC) summarize the overall trend across the study period. Rates are expressed per 100,000 population and age-adjusted to the 2000 U.S. standard population.

### Opposite AAMR trajectories for E10- and E11- recorded deaths

Analyses based on recorded E10 and E11 mentions showed opposite mortality trajectories. E10-nonUCD AAMRs declined broadly across major underlying-cause categories examined. The decline was most pronounced for circulatory disease-related mortality, which decreased from 2.74 to 0.34 per 100,000 (AAPC, -7.79%; 95% CI, -8.11 to -7.52) (**Supplementary Figure 2A**). Marked declines were also observed for malignant neoplasms, respiratory diseases, infectious diseases, nervous system diseases, and digestive diseases (**Supplementary Figures 2B–F**).

In contrast, E11-nonUCD AAMRs increased across the major underlying-cause categories examined. Circulatory disease-related E11-nonUCD mortality increased from 5.87 to 10.25 per 100,000 (AAPC, 2.20%; 95% CI, 1.93 to 2.58), showing that the cardiovascular burden did not disappear despite the declining circulatory share (**Figure 2A**). The steepest increases were observed for mental and behavioral disorders (AAPC, 10.31%; 95% CI, 9.40 to 12.27) and diseases of the nervous system (AAPC, 7.50%; 95% CI, 7.16 to 8.03) (**Figures 2B, C**). Malignant neoplasm-related, respiratory disease-related, digestive disease-related, and circulatory disease-related AAMRs also increased for E11 (**Figures 2D–F**). The model-setting sensitivity analyses produced results that were consistent with the main long-term trends observed in the primary analyses (**Supplementary File**).

**Figure 2:**
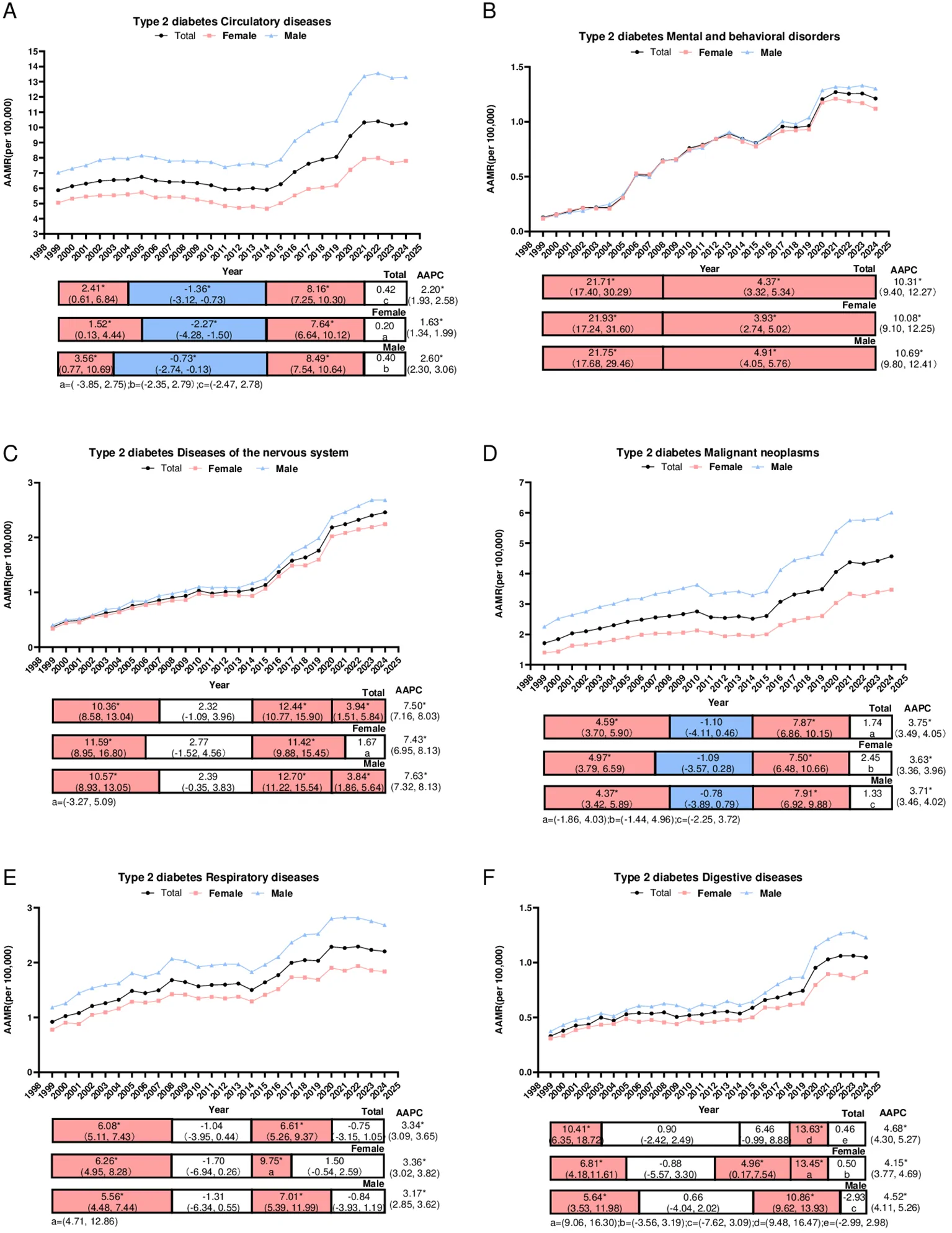
Age-adjusted mortality trends for selected underlying causes among E11-nonUCD deaths, United States, 1999–2024. Annual age-adjusted mortality rates (AAMR) for underlying causes assigned to **(A)** circulatory diseases, **(B)** mental and behavioral disorders, **(C)** diseases of the nervous system, **(D)** malignant neoplasms, **(E)** respiratory diseases, and **(F)** digestive diseases. Total estimates are shown together with sex-specific estimates for reference. Joinpoint segment bars below each plot indicate annual percentage changes (APC) for each fitted segment. Average annual percentage changes (AAPC) summarize the overall trend across the study period. Rates are expressed per 100,000 population and age-adjusted to the 2000 U.S. standard population.

### Circulatory disease subtypes

Within circulatory DM-nonUCD deaths, ischemic heart disease was the largest subtype, accounting for 1,317,463 deaths, or 27.48% of all DM-nonUCD deaths (A similar decline in the circulatory share was observed for both E10 and E111). Cerebrovascular disease, heart failure, hypertensive disease, and peripheral vascular disease were the next most common circulatory subtypes. The proportional profile shifted away from ischemic heart disease over time. The share of all DM-nonUCD deaths attributed to ischemic heart disease decreased from 39.03% in 1999 to 22.21% in 2024 (**Supplementary Table 11**). In contrast, hypertensive disease increased from 3.66% to 7.67%, and heart failure remained persistently common (**Supplementary Table 11**).

AAMR and Joinpoint analyses confirmed different trajectories across circulatory subtypes. Ischemic heart disease-related DM-nonUCD mortality declined from 20.18 to 12.31 per 100,000 (AAPC, -2.04%; 95% CI, -2.23 to -1.92) (**Figure 3A**). Cerebrovascular disease-related mortality also declined from 4.87 to 3.52 per 100,000 (AAPC, -1.43%; 95% CI, -1.76 to -1.21) (**Figure 3B**). In contrast, hypertensive disease-related mortality increased from 1.90 to 4.32 per 100,000 (AAPC, 3.21%; 95% CI, 2.94 to 3.56) (**Figure 3C**). These findings indicate that the decline in circulatory DM-nonUCD mortality was driven mainly by ischemic heart disease and cerebrovascular disease, whereas hypertensive disease became more prominent.

**Figure 3:**
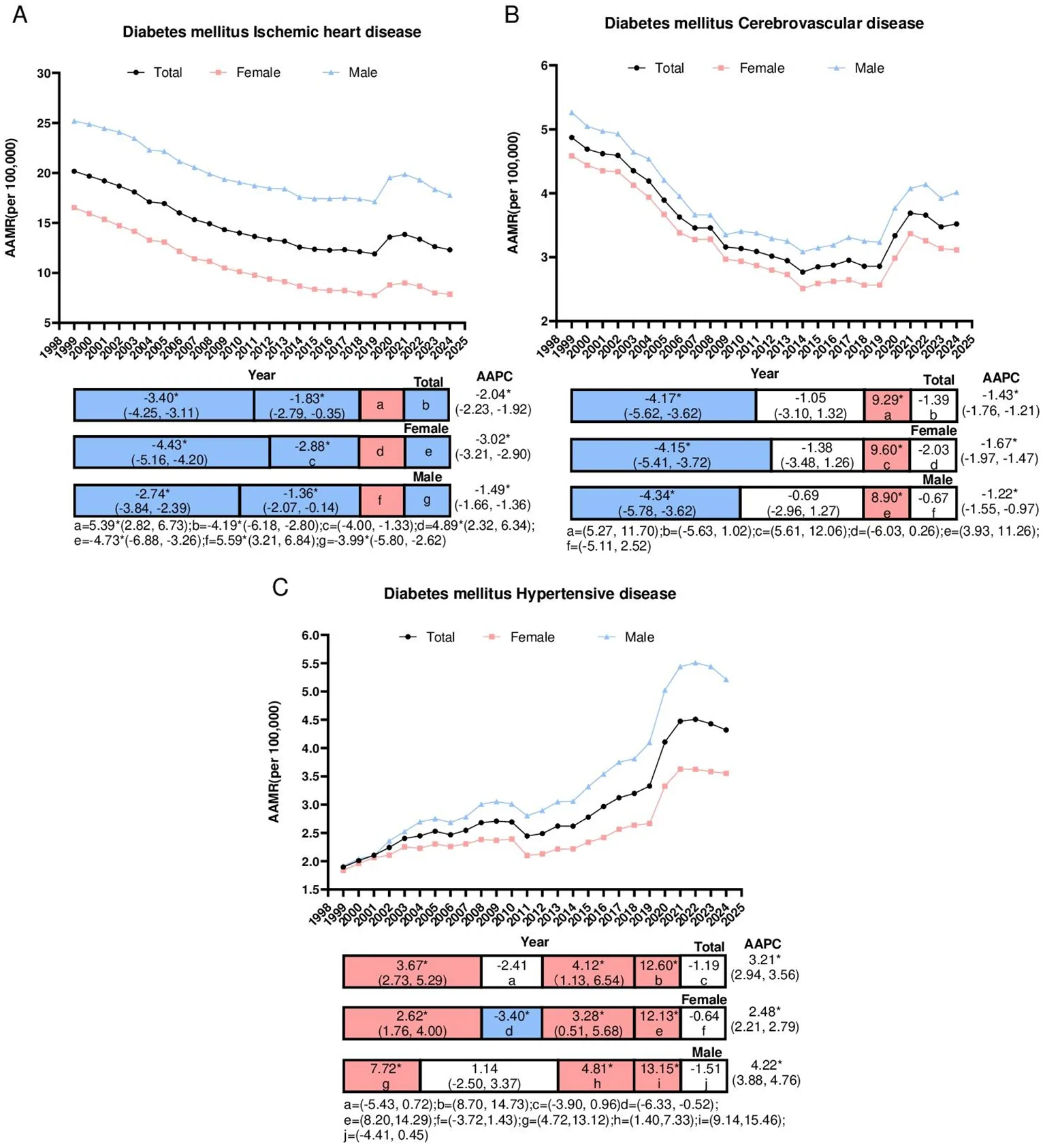
Age-adjusted mortality trends for selected circulatory disease subtypes among DM-nonUCD deaths, United States, 1999–2024. Annual age-adjusted mortality rates (AAMR) for underlying causes assigned to **(A)** ischemic heart disease, **(B)** cerebrovascular disease, and **(C)** hypertensive disease. Total estimates are shown together with sex-specific estimates for reference. Joinpoint segment bars below each plot indicate annual percentage changes (APC) for each fitted segment. Average annual percentage changes (AAPC) summarize the overall trend across the study period. Rates are expressed per 100,000 population and age-adjusted to the 2000 U.S. standard population. DM-nonUCD, diabetes recorded as a non-underlying cause of death.

Circulatory trends also differed between deaths with recorded E10 and E11 mentions. Among E10-nonUCD deaths, ischemic heart disease, hypertensive disease, and cerebrovascular disease AAMRs all declined (**Figures 4A–C**). Among E11-nonUCD deaths, the same circulatory subtypes all increased. The increase was largest for hypertensive disease (AAPC, 6.47%; 95% CI, 6.13 to 6.98), followed by cerebrovascular disease (AAPC, 2.04%; 95% CI, 1.65 to 2.57) and ischemic heart disease (AAPC, 1.06%; 95% CI, 0.81 to 1.39) (**Figures 4D–F**).

**Figure 4:**
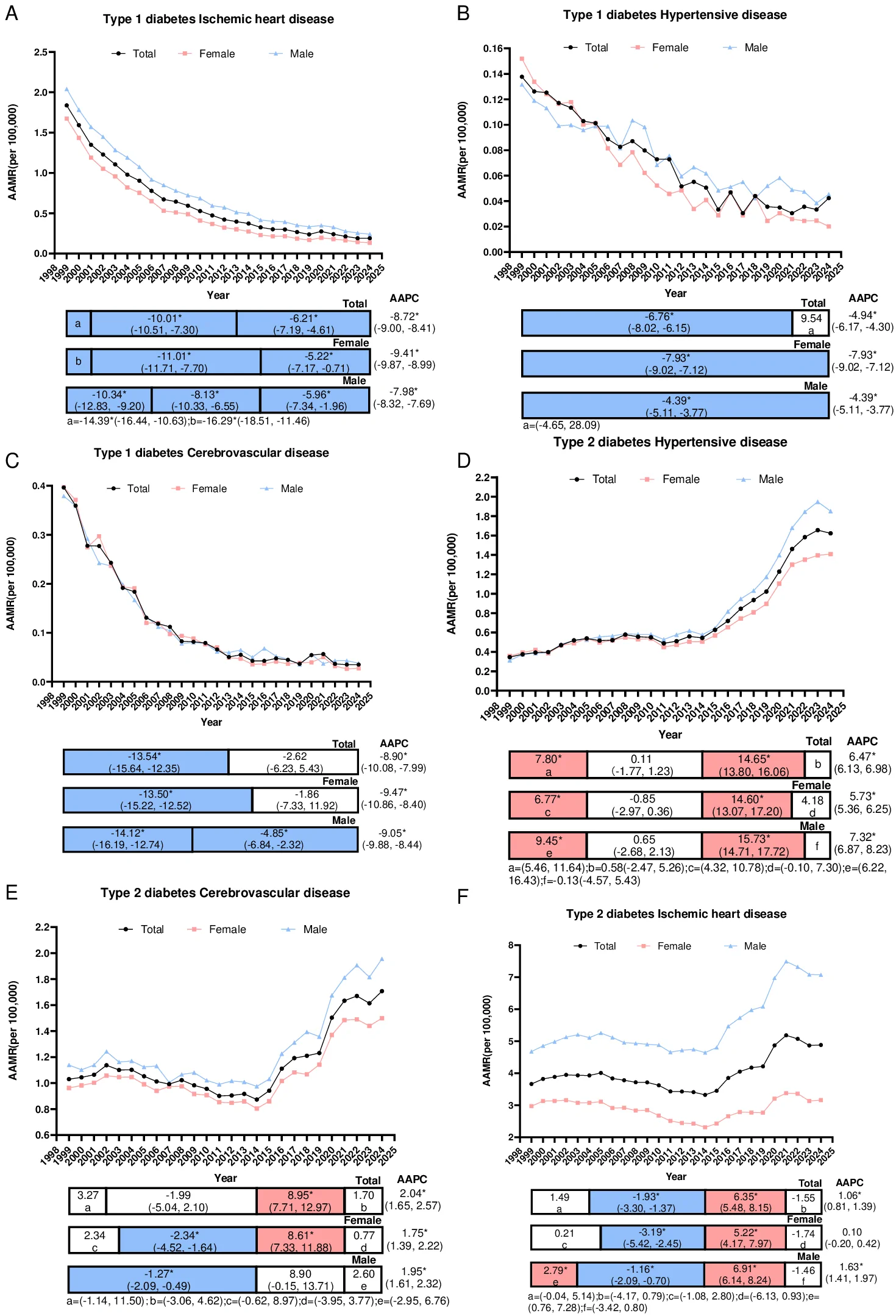
Trends in selected circulatory disease-related mortality by recorded E10 and E11 mentions, United States, 1999–2024. **(A–C)** Annual age-adjusted mortality rates (AAMR) for selected circulatory disease subtypes among E10-nonUCD deaths. **(D–F)** Corresponding trends among E11-nonUCD deaths. Total estimates are shown together with sex-specific estimates for reference. Joinpoint segment bars below each plot indicate annual percentage changes (APC) for each fitted segment. Average annual percentage changes (AAPC) summarize the overall trend across the study period. Rates are expressed per 100,000 population and age-adjusted to the 2000 U.S. standard population. E10-nonUCD, type 1 diabetes recorded as a non-underlying cause of death; E11-nonUCD, type 2 diabetes recorded as a non-underlying cause of death.

### Sex-specific mortality trends

Sex-specific trends were generally consistent with the overall findings. Among DM-nonUCD deaths, circulatory disease-related mortality declined more rapidly in women than in men (AAPC, −1.64% vs. −0.59%), whereas mortality related to mental and behavioral disorders and diseases of the nervous system increased in both sexes. Declines in ischemic heart disease and cerebrovascular disease were also greater in women, while hypertensive disease-related mortality increased more rapidly in men (AAPC, 4.22% vs. 2.48%). Cause-specific E10-nonUCD mortality generally declined in both sexes, whereas the corresponding E11-nonUCD trends increased (**Figure 2**; **Supplementary Figure 2**).

### 1999–2019 pre-pandemic sensitivity analysis

The 1999–2019 sensitivity analysis showed that the redistribution pattern was already evident before the COVID-19 pandemic. In overall DM-nonUCD deaths, circulatory disease-related AAMR declined, whereas AAMRs for diseases of the nervous system and mental and behavioral disorders increased (**Supplementary Figures 3A–F**). For E11-nonUCD, AAMRs increased in all three selected categories, with AAPCs of 3.22% for circulatory diseases, 7.38% for diseases of the nervous system, and 4.44% for mental and behavioral disorders (**Supplementary Figures 4A–F**). E10-nonUCD showed less consistent patterns; mental and behavioral disorder-related AAMR had no significant overall AAPC despite segment-specific changes (**Supplementary Figures 5A–F**). Sex-specific estimates from the pre-pandemic analyses were consistent with these overall patterns (**Supplementary Figures 3**–**5**).

## Discussion

In this national multiple-cause-of-death analysis, diabetes recorded as a non-underlying cause identified nearly 4.8 million U.S. deaths from 1999 through 2024 that would not be counted as diabetes underlying-cause deaths. The underlying-cause profile changed substantially over time. Circulatory diseases remained the leading category, followed by malignant neoplasms and respiratory diseases. Within the circulatory category, ischemic heart disease accounted for much of the decline, whereas hypertensive disease became more prominent. At the same time, malignant neoplasms, diseases of the nervous system, and mental and behavioral disorders accounted for increasing shares of DM-nonUCD deaths. The recorded diabetes subtype profile also changed, with E11-nonUCD deaths increasing markedly and E10-nonUCD deaths declining. These patterns suggest that DM-nonUCD increasingly reflects deaths occurring in the setting of diabetes and multimorbidity, rather than deaths dominated by ischemic heart disease alone.

The decline in the proportional contribution of circulatory disease should not be interpreted as a parallel decline in cardiovascular mortality burden. Circulatory disease remained the leading underlying-cause category throughout the study period, and circulatory mortality increased among E11-nonUCD deaths despite declining overall for E10–E14. Among E11-nonUCD deaths, cardiovascular mortality therefore remained important but became less dominant as other competing causes increased. Within circulatory disease, the overall decline was driven largely by ischemic heart disease and cerebrovascular disease, consistent with previous reports of declining acute cardiovascular complications among people with diabetes (12). By contrast, hypertensive disease-related mortality increased, while heart failure remained an important contributor. The rise in hypertensive disease should be interpreted cautiously, as it may reflect both a genuine increase in hypertension-related mortality and secular changes in clinical recognition, death certification, and causal sequencing. U.S. data have documented temporal changes and errors in the ordering of hypertension and diabetes on death certificates (13), and similar certification effects may influence coronary heart disease mortality estimates (14), although the relative contributions of true disease trends and certification changes cannot be disentangled in CDC WONDER. Together, these findings suggest that diabetes-related cardiovascular mortality surveillance should consider hypertensive disease and heart failure in addition to ischemic heart disease (6, 8, 15).

The divergent trajectories of E10- and E11-nonUCD mortality were another major finding. E11-nonUCD deaths increased substantially, with rising AAMRs across the major underlying-cause categories examined, whereas E10-nonUCD mortality declined broadly. These contrasting patterns suggest that the aggregate E10–E14 trend should not be interpreted as a single diabetes trajectory. The increase in recorded E11-nonUCD may reflect several concurrent factors, including the expanding population living with type 2 diabetes, population aging, longer survival with chronic cardiometabolic disease, and secular changes in diabetes subtype documentation and death-certificate coding (2, 16). This interpretation is supported by the growing global burden of type 2 diabetes (2) and persistent excess mortality among affected populations despite advances in care (16). Conversely, the decline in E10-nonUCD mortality may reflect improvements in type 1 diabetes management, including intensive glycemic control and prevention of cardiovascular and renal complications (5, 17, 18). This interpretation is consistent with DCCT/EDIC evidence showing long-term cardiovascular benefit after intensive therapy in type 1 diabetes (19) and lower long-term mortality after early intensive treatment (20). However, because subtype classification was based on death-certificate coding rather than validated clinical diagnoses, part of the observed divergence may also reflect changes in coding practice, including more specific assignment of E11 and potential misclassification of diabetes type. These findings should therefore be interpreted as distinct recorded mortality trajectories rather than direct measures of the true population burden of type 1 and type 2 diabetes.

Population aging likely contributed to the changing underlying-cause profile but does not appear to fully explain the observed redistribution. The circulatory share declined in both age groups, whereas the increasing contribution of mental and behavioral disorders and diseases of the nervous system was more pronounced among those aged ≥65 years. Age therefore appears to have influenced the magnitude rather than the direction of these changes. This pattern is consistent with broader evidence that diabetes is associated with both vascular and nonvascular mortality (3). It also parallels longitudinal data from England showing declining vascular mortality alongside increasing contributions from dementia, liver disease, and cancer among people with diabetes over time (4). Our findings extend this literature by demonstrating a similar transition in U.S. deaths in which diabetes was recorded as clinically relevant but was not selected as the underlying cause.

The increasing contribution of nervous system diseases and mental and behavioral disorders likely reflects, at least in part, the growing prominence of dementia-related mortality. U.S. mortality data have documented substantial increases in Alzheimer disease mortality over time, a pattern attributed to population aging, improved diagnosis and reporting, and declining mortality from competing causes such as cardiovascular disease and stroke (21). Our findings are consistent with this broader transition. As mortality from ischemic heart disease and cerebrovascular disease becomes less dominant, older adults with recorded diabetes may survive to ages at which neurodegenerative, cognitive, and frailty-related conditions are more likely to be selected as the underlying cause of death. This interpretation should remain cautious, however, because death-certificate data cannot distinguish true changes in disease occurrence from changes in diagnosis, recognition, or certification practice.

The increasing contribution of malignant neoplasms and respiratory diseases should not be interpreted as evidence of direct causation by diabetes. Diabetes may share biological and behavioral pathways with several nonvascular conditions, including obesity, insulin resistance, chronic inflammation, renal impairment, immune dysfunction, and common lifestyle risk factors (3, 4). However, death-certificate data cannot determine whether diabetes caused, accelerated, complicated, or merely coexisted with the assigned underlying cause. Importantly, the 1999–2019 sensitivity analysis showed that the broader redistribution of mortality was already evident before the COVID-19 pandemic, indicating that COVID-19 represented a major pandemic-era disruption but did not create the long-term pattern (22, 23). From a surveillance and health-system perspective, these findings support a broader view of diabetes-related mortality that extends beyond glycemic control and ischemic cardiovascular disease, with greater attention to multimorbidity and competing cardiovascular, oncologic, respiratory, and neurocognitive conditions.

Several limitations should be considered. First, death-certificate data are subject to underreporting and misclassification, and secular changes in diagnostic recognition, causal sequencing, and certification practices may have influenced both whether diabetes was recorded and which condition was selected as the underlying cause (24–26). These changes may also have affected the attribution of specific cardiovascular causes, including hypertension and coronary disease (13, 14). Second, diabetes subtype coding may be inaccurate. The E10- and E11-specific analyses were based on recorded ICD-10 mentions rather than validated clinical diagnoses; these categories should therefore not be interpreted as mutually exclusive clinical cohorts. Some deaths may have been coded as E11 or unspecified diabetes despite a different clinical diabetes type. Third, DM-nonUCD indicates that diabetes was recorded on the death certificate but not selected as the underlying cause and should not be interpreted as evidence that diabetes causally contributed to death. Fourth, CDC WONDER provides aggregated mortality data and lacks information on diabetes duration, glycemic control, body mass index, kidney function, medication use, socioeconomic position, health-care access, and other clinical comorbidities. Accordingly, the reported rates represent population-level mortality rates per 100,000 U.S. residents rather than mortality risks among people living with diabetes. Finally, mortality patterns during 2020–2024 were substantially affected by COVID-19; pandemic-era estimates should therefore be interpreted as part of the contemporary mortality profile rather than as a simple extension of pre-pandemic trends.

In conclusion, DM-nonUCD represents a large and changing component of U.S. mortality. From 1999 to 2024, the recorded DM-nonUCD profile changed, with an increasing proportion of E11 mentions, a declining proportion of E10 mentions, and a wider distribution of competing underlying causes. The circulatory profile also changed, with declining ischemic heart disease and cerebrovascular disease but a growing contribution from hypertensive disease and a persistent contribution from heart failure. These findings support reporting DM-nonUCD alongside diabetes underlying-cause mortality and diabetes mentioned-anywhere mortality. They also suggest that surveillance and care planning for older adults and patients with long-duration type 2 diabetes should account for competing risks beyond glycemic control and ischemic heart disease prevention, including hypertensive disease, heart failure, cognitive and mental health conditions, respiratory disease, infection-related mortality, and cancer.

## Data Availability

Data for this study were obtained from the publicly available CDC WONDER Multiple Cause of Death database (https://wonder.cdc.gov/mcd.html). The analysis was based on de-identified, aggregated mortality data, and no individual-level participant data were accessed. ICD-10 codes, case definitions, and analytic methods are described in the Methods. Derived aggregate data supporting the tables and figures are available from the corresponding author XH (Email:hongxuezhi8@126.com) upon reasonable request.
