# Supplementary material for: Shifting competing underlying causes of death among U.S. decedents with diabetes recorded as a non-underlying cause, 1999–2024
Source: Front Endocrinol (Lausanne). 2026 Sep 30;17:1957392. doi: 10.3389/fendo.2026.1957392 (PMC13661304; doi:10.3389/fendo.2026.1957392)
Supplement: Supplementary file 1 [file DataSheet1.docx]

**Supplementary materials**

Supplementary Table 1. CDC WONDER query settings used for data extraction.

Supplementary Table 2. ICD-10 code definitions used for diabetes ascertainment, competing underlying-cause classification, and circulatory disease subtype analysis.

Supplementary Table 3. Annual counts and underlying-cause distributions of DM-nonUCD deaths, United States, 1999–2024.

Supplementary Table 4. Annual numbers of DM-nonUCD deaths and their proportion among all U.S. deaths, 1999–2024

Supplementary Table 5. Annual counts and underlying-cause distributions of E10-nonUCD deaths, United States, 1999–2024.

Supplementary Table 6. Annual counts and underlying-cause distributions of E11-nonUCD deaths, United States, 1999–2024.

Supplementary Table 7. Period-specific underlying-cause distribution of E10-nonUCD deaths, United States, 1999–2024.

Supplementary Table 8. Distribution of underlying causes among deaths with diabetes (E10–E14) recorded as a non-underlying cause, by age group and study period, 1999–2024.

Supplementary Table 9. Distribution of underlying causes among deaths with diabetes (E11) recorded as a non-underlying cause, by age group and study period, 1999–2024.

Supplementary Table 10. Distribution of underlying causes among deaths with diabetes (E10) recorded as a non-underlying cause, by age group and study period, 1999–2024.

Supplementary Table 11. Annual counts and proportions of selected circulatory disease subtypes among DM-nonUCD, E10-nonUCD, and E11-nonUCD deaths, United States, 1999–2024.

Supplementary Figure 1. Annual burden and recorded diabetes subtype composition of DM-nonUCD deaths, United States, 1999–2024.

Supplementary Figure 2. Age-adjusted mortality trends for selected underlying causes among E10-nonUCD deaths, United States, 1999–2024.

Supplementary Figure 3. Pre-pandemic age-adjusted mortality trends for selected underlying causes among DM-nonUCD deaths, United States, 1999–2019.

Supplementary Figure 4. Pre-pandemic age-adjusted mortality trends for selected underlying causes among E11-nonUCD deaths, United States, 1999–2019.

Supplementary Figure 5. Pre-pandemic age-adjusted mortality trends for selected underlying causes among E10-nonUCD deaths, United States, 1999–2019.

**Supplementary Table 1. CDC WONDER query settings used for data extraction**

| **Query component** | **Setting used in this study** |
| --- | --- |
| Data source | CDC WONDER Multiple Cause of Death databases |
| Study population | U.S. resident deaths |
| Study period | 1999–2024 |
| Grouping variables | Year for the overall analysis; Year and Sex for the sex-stratified analysis; and Year and Age Groups for the age-stratified analysis |
| Underlying cause of death | Each underlying-cause category was selected separately using the corresponding ICD-10 codes listed in Supplementary Table 2 |
| Multiple cause of death | E10–E14 for the overall diabetes analysis, E10 for the type 1 diabetes analysis, and E11 for the type 2 diabetes analysis |
| Measures selected | Death counts, and age-adjusted mortality rates, together with their 95% confidence intervals and standard errors, where available |
| Data extraction method | Separate aggregate queries were performed for each underlying-cause category through the CDC WONDER web interface; no custom data-extraction code was used |
| Standard population | 2000 U.S. standard population |

**Supplementary Table 2. ICD-10 code definitions used for diabetes ascertainment, competing underlying-cause classification, and circulatory disease subtype analysis**

| Category | ICD-10 code(s) | Table/Figure label |
| --- | --- | --- |
| Diabetes mellitus | E10–E14 | Diabetes mellitus |
| Type 1 diabetes mellitus | E10 | Type 1 diabetes |
| Type 2 diabetes mellitus | E11 | Type 2 diabetes |
| Certain infectious and parasitic diseases | A00–B99 | Infectious and parasitic diseases |
| Malignant neoplasms | C00–C97 | Malignant neoplasms |
| In situ, benign, and uncertain/unknown behavior neoplasms | D00–D48 | Other neoplasms |
| Diseases of the blood and blood-forming organs and certain immune disorders | D50–D89 | Blood/immune diseases |
| Endocrine, nutritional, and metabolic diseases other than diabetes mellitus | E00–E09, E15–E88 | Other endocrine/metabolic diseases |
| Mental and behavioural disorders | F01–F99 | Mental and behavioral disorders |
| Diseases of the nervous system | G00–G98 | Diseases of the nervous system |
| Diseases of the circulatory system | I00–I99 | Circulatory diseases |
| Hypertensive diseases | I10–I15 | Hypertensive disease |
| Ischaemic heart diseases | I20–I25 | Ischemic heart disease |
| Peripheral vascular disease / diseases of arteries, arterioles, and capillaries | I70–I79 | Peripheral vascular disease |
| Heart failure | I50 | Heart failure |
| Cerebrovascular diseases | I60–I69 | Cerebrovascular disease |
| Diseases of the respiratory system | J00–J98 | Respiratory diseases |
| Diseases of the digestive system | K00–K92 | Digestive diseases |
| Diseases of the skin and subcutaneous tissue | L00–L98 | Skin/subcutaneous diseases |
| Diseases of the musculoskeletal system and connective tissue | M00–M99 | Musculoskeletal/connective tissue diseases |
| Diseases of the genitourinary system | N00–N98 | Genitourinary diseases |
| Congenital malformations, deformations, and chromosomal abnormalities | Q00–Q99 | Congenital/chromosomal conditions |
| COVID-19 | U07.1 | COVID-19 |

**Supplementary Table 3. Annual burden, recorded diabetes subtype composition, and underlying-cause distribution of DM-nonUCD, United States,** **1999–2024.**

| **Year** | **nonUCD (n)** | **A00–B99 n(%)** | **C00–C97 n(%)** | **D00-D48 n(%)** | **D50-D89 n(%)** | **E00–E09, E15–E88 n(%)** | **F01–F99 n(%)** | **G00–G98 n(%)** | **I00–I99 n(%)** | **J00–J98 n(%)** | **K00–K92 n(%)** | **L00-L98 n(%)** | **M00-M99 n(%)** | **N00–N98 n(%)** | **Q00-Q99 n(%)** | **U07.1 n(%)** |
| --- | --- | --- | --- | --- | --- | --- | --- | --- | --- | --- | --- | --- | --- | --- | --- | --- |
| **DM (E10-E14)** | | | | | | | | | | | | | | | | |
| 1999 | 141371 | 3695(2.61%) | 19810(14.01%) | 617(0.44%) | 431(0.30%) | 1781(1.26%) | 1483(1.05%) | 3772(2.67%) | 85547(60.51%) | 11782(8.33%) | 4769(3.37%) | 197(0.14%) | 880(0.62%) | 4353(3.08%) | 134(0.09%) |  |
| 2000 | 143763 | 3458(2.41%) | 20301(14.12%) | 660(0.46%) | 526(0.37%) | 1981(1.38%) | 1658(1.15%) | 4220(2.94%) | 84910(59.06%) | 12915(8.98%) | 5071(3.53%) | 214(0.15%) | 965(0.67%) | 4499(3.13%) | 152(0.11%) |  |
| 2001 | 146755 | 3797(2.59%) | 21308(14.52%) | 695(0.47%) | 545(0.37%) | 2006(1.37%) | 1944(1.32%) | 4577(3.12%) | 85144(58.02%) | 12912(8.80%) | 5418(3.69%) | 247(0.17%) | 994(0.68%) | 4653(3.17%) | 128(0.09%) |  |
| 2002 | 150843 | 4086(2.71%) | 22182(14.71%) | 696(0.46%) | 581(0.39%) | 2196(1.46%) | 2231(1.48%) | 4997(3.31%) | 85539(56.71%) | 14036(9.31%) | 5422(3.59%) | 274(0.18%) | 1101(0.73%) | 4766(3.16%) | 157(0.10%) |  |
| 2003 | 153357 | 4289(2.80%) | 22877(14.92%) | 739(0.48%) | 545(0.36%) | 2351(1.53%) | 2360(1.54%) | 5683(3.71%) | 85077(55.48%) | 14226(9.28%) | 5734(3.74%) | 319(0.21%) | 1156(0.75%) | 4997(3.26%) | 159(0.10%) |  |
| 2004 | 152313 | 4272(2.80%) | 23233(15.25%) | 762(0.50%) | 565(0.37%) | 2462(1.62%) | 2428(1.59%) | 5920(3.89%) | 82996(54.49%) | 14351(9.42%) | 5660(3.72%) | 329(0.22%) | 1194(0.78%) | 4940(3.24%) | 147(0.10%) |  |
| 2005 | 158499 | 4648(2.93%) | 24231(15.29%) | 760(0.48%) | 586(0.37%) | 2652(1.67%) | 3142(1.98%) | 6561(4.14%) | 83830(52.89%) | 15876(10.02%) | 5917(3.73%) | 335(0.21%) | 1189(0.75%) | 5291(3.34%) | 146(0.09%) |  |
| 2006 | 158588 | 4540(2.86%) | 24645(15.54%) | 830(0.52%) | 529(0.33%) | 2623(1.65%) | 5556(3.50%) | 7055(4.45%) | 80927(51.03%) | 15325(9.66%) | 5965(3.76%) | 522(0.33%) | 1241(0.78%) | 5171(3.26%) | 169(0.11%) |  |
| 2007 | 160020 | 4656(2.91%) | 25339(15.83%) | 799(0.50%) | 714(0.45%) | 2745(1.72%) | 5496(3.43%) | 7335(4.58%) | 80185(50.11%) | 15745(9.84%) | 5949(3.72%) | 525(0.33%) | 1215(0.76%) | 5161(3.23%) | 152(0.09%) |  |
| 2008 | 163276 | 4595(2.81%) | 25435(15.58%) | 877(0.54%) | 671(0.41%) | 2864(1.75%) | 6607(4.05%) | 7899(4.84%) | 80320(49.19%) | 17311(10.60%) | 6002(3.68%) | 504(0.31%) | 1206(0.74%) | 4690(2.87%) | 163(0.10%) |  |
| 2009 | 161937 | 4666(2.88%) | 26078(16.10%) | 853(0.53%) | 611(0.38%) | 3006(1.86%) | 6683(4.13%) | 7988(4.93%) | 78795(48.66%) | 16942(10.46%) | 5797(3.58%) | 472(0.29%) | 1191(0.74%) | 4491(2.77%) | 132(0.08%) |  |
| 2010 | 164980 | 4767(2.89%) | 26758(16.22%) | 863(0.52%) | 611(0.37%) | 3096(1.88%) | 7626(4.62%) | 8640(5.24%) | 79041(47.91%) | 16624(10.08%) | 5874(3.56%) | 469(0.28%) | 1233(0.75%) | 4648(2.82%) | 180(0.11%) |  |
| 2011 | 165358 | 4758(2.88%) | 26742(16.17%) | 868(0.52%) | 692(0.42%) | 3282(1.98%) | 8660(5.24%) | 8978(5.43%) | 78776(47.64%) | 17645(10.67%) | 6016(3.64%) | 532(0.32%) | 1324(0.80%) | 2024(1.22%) | 158(0.10%) |  |
| 2012 | 167175 | 4662(2.79%) | 27106(16.21%) | 863(0.52%) | 703(0.42%) | 3301(1.97%) | 9506(5.69%) | 9049(5.41%) | 79598(47.61%) | 17371(10.39%) | 6077(3.64%) | 520(0.31%) | 1287(0.77%) | 1934(1.16%) | 173(0.10%) |  |
| 2013 | 171226 | 4880(2.85%) | 27544(16.09%) | 871(0.51%) | 712(0.42%) | 3378(1.97%) | 9998(5.84%) | 9211(5.38%) | 81357(47.51%) | 18015(10.52%) | 6282(3.67%) | 531(0.31%) | 1120(0.65%) | 1980(1.16%) | 197(0.12%) |  |
| 2014 | 168528 | 4478(2.66%) | 26672(15.83%) | 846(0.50%) | 693(0.41%) | 3480(2.06%) | 9603(5.70%) | 9492(5.63%) | 80205(47.59%) | 17403(10.33%) | 6199(3.68%) | 521(0.31%) | 1123(0.67%) | 2000(1.19%) | 176(0.10%) |  |
| 2015 | 173271 | 4665(2.69%) | 26955(15.56%) | 906(0.52%) | 736(0.42%) | 3829(2.21%) | 8976(5.18%) | 10499(6.06%) | 82365(47.54%) | 18031(10.41%) | 6459(3.73%) | 533(0.31%) | 1241(0.72%) | 2126(1.23%) | 185(0.11%) |  |
| 2016 | 178794 | 4660(2.61%) | 28214(15.78%) | 900(0.50%) | 738(0.41%) | 4009(2.24%) | 8651(4.84%) | 11428(6.39%) | 84764(47.41%) | 18008(10.07%) | 6859(3.84%) | 569(0.32%) | 1268(0.71%) | 2240(1.25%) | 190(0.11%) |  |
| 2017 | 187138 | 4740(2.53%) | 29159(15.58%) | 880(0.47%) | 753(0.40%) | 4211(2.25%) | 9181(4.91%) | 12244(6.54%) | 88377(47.23%) | 19441(10.39%) | 6931(3.70%) | 565(0.30%) | 1240(0.66%) | 2251(1.20%) | 202(0.11%) |  |
| 2018 | 190983 | 4672(2.45%) | 29788(15.60%) | 909(0.48%) | 762(0.40%) | 4452(2.33%) | 9189(4.81%) | 13034(6.82%) | 89211(46.71%) | 19807(10.37%) | 7248(3.80%) | 637(0.33%) | 1342(0.70%) | 2354(1.23%) | 204(0.11%) |  |
| 2019 | 195154 | 4450(2.28%) | 30837(15.80%) | 942(0.48%) | 705(0.36%) | 4767(2.44%) | 9381(4.81%) | 13687(7.01%) | 90804(46.53%) | 19426(9.95%) | 7357(3.77%) | 671(0.34%) | 1436(0.74%) | 2422(1.24%) | 229(0.12%) |  |
| 2020 | 285878 | 5429(1.90%) | 35269(12.34%) | 1074(0.38%) | 921(0.32%) | 5971(2.09%) | 11267(3.94%) | 16738(5.85%) | 106616(37.29%) | 21925(7.67%) | 8978(3.14%) | 743(0.26%) | 1734(0.61%) | 2945(1.03%) | 234(0.08%) | 56610(19.80%) |
| 2021 | 296107 | 5576(1.88%) | 37480(12.66%) | 1077(0.36%) | 924(0.31%) | 6760(2.28%) | 10889(3.68%) | 16580(5.60%) | 110029(37.16%) | 20852(7.04%) | 9893(3.34%) | 883(0.30%) | 1994(0.67%) | 3276(1.11%) | 290(0.10%) | 58920(19.90%) |
| 2022 | 268244 | 5948(2.22%) | 38601(14.39%) | 1150(0.43%) | 985(0.37%) | 7095(2.64%) | 11406(4.25%) | 17685(6.59%) | 112455(41.92%) | 21877(8.16%) | 10356(3.86%) | 938(0.35%) | 2101(0.78%) | 3222(1.20%) | 285(0.11%) | 23142(8.63%) |
| 2023 | 244152 | 5823(2.38%) | 38849(15.91%) | 1137(0.47%) | 932(0.38%) | 6802(2.79%) | 10959(4.49%) | 17833(7.30%) | 108320(44.37%) | 20865(8.55%) | 9996(4.09%) | 931(0.38%) | 2031(0.83%) | 3178(1.30%) | 318(0.13%) | 5302(2.17%) |
| 2024 | 246426 | 5793(2.35%) | 40179(16.30%) | 1088(0.44%) | 993(0.40%) | 7090(2.88%) | 10879(4.41%) | 18623(7.56%) | 109611(44.48%) | 21126(8.57%) | 10151(4.12%) | 876(0.36%) | 2150(0.87%) | 3048(1.24%) | 315(0.13%) | 3197(1.30%) |
| Total | 4794136 | 122003(2.54%) | 725592(15.13%) | 22662(0.47%) | 18164(0.38%) | 98190(2.05%) | 185759(3.87%) | 259728(5.42%) | 2294799(47.87%) | 449837(9.38%) | 176380(3.68%) | 13857(0.29%) | 34956(0.73%) | 92660(1.93%) | 4975(0.10%) | 147171(3.07%) |

**Supplementary Table 4. Annual numbers of DM-nonUCD deaths and their proportion among all U.S. deaths, 1999–2024.**

| Year | Deaths | DM-nonUCD | Percentage(%) |
| --- | --- | --- | --- |
| 1999 | 2391399 | 141371 | 5.91 |
| 2000 | 2403351 | 143763 | 5.98 |
| 2001 | 2416425 | 146755 | 6.07 |
| 2002 | 2443387 | 150843 | 6.17 |
| 2003 | 2448288 | 153357 | 6.26 |
| 2004 | 2397615 | 152313 | 6.35 |
| 2005 | 2448017 | 158499 | 6.47 |
| 2006 | 2426264 | 158588 | 6.54 |
| 2007 | 2423712 | 160020 | 6.60 |
| 2008 | 2471984 | 163276 | 6.61 |
| 2009 | 2437163 | 161937 | 6.64 |
| 2010 | 2468435 | 164980 | 6.68 |
| 2011 | 2515458 | 165358 | 6.57 |
| 2012 | 2543279 | 167175 | 6.57 |
| 2013 | 2596993 | 171226 | 6.59 |
| 2014 | 2626418 | 168528 | 6.42 |
| 2015 | 2712630 | 173271 | 6.39 |
| 2016 | 2744248 | 178794 | 6.52 |
| 2017 | 2813503 | 187138 | 6.65 |
| 2018 | 2839205 | 190983 | 6.73 |
| 2019 | 2854838 | 195154 | 6.84 |
| 2020 | 3383729 | 285878 | 8.45 |
| 2021 | 3464231 | 296107 | 8.55 |
| 2022 | 3279857 | 268244 | 8.18 |
| 2023 | 3090964 | 244152 | 7.90 |
| 2024 | 3072666 | 246426 | 8.02 |

**Supplementary Table 5. Annual burden, recorded diabetes subtype composition, and underlying-cause distribution of E10-nonUCD, United States, 1999–2024.**

| **Year** | **nonUCD (n)** | **A00–B99 n(%)** | **C00–C97 n(%)** | **D00-D48 n(%)** | **D50-D89 n(%)** | **E00–E09, E15–E88 n(%)** | **F01–F99 n(%)** | **G00–G98 n(%)** | **I00–I99 n(%)** | **J00–J98 n(%)** | **K00–K92 n(%)** | **L00-L98 n(%)** | **M00-M99 n(%)** | **N00–N98 n(%)** | **Q00-Q99 n(%)** | **U07.1 n(%)** |
| --- | --- | --- | --- | --- | --- | --- | --- | --- | --- | --- | --- | --- | --- | --- | --- | --- |
| **E10** | | | | | | | | | | | | | | | | |
| 1999 | 12438 | 359(2.89%) | 1608(12.93%) | 55(0.44%) | 52(0.42%) | 153(1.23%) | 126(1.01%) | 332(2.67%) | 7500(60.30%) | 1072(8.62%) | 384(3.09%) | 17(0.14%) | 92(0.74%) | 468(3.76%) | 16(0.13%) |  |
| 2000 | 11162 | 289(2.59%) | 1474(13.21%) | 45(0.40%) | 43(0.39%) | 158(1.42%) | 116(1.04%) | 325(2.91%) | 6628(59.38%) | 973(8.72%) | 373(3.34%) | 16(0.14%) | 75(0.67%) | 410(3.67%) | 14(0.13%) |  |
| 2001 | 10069 | 310(3.08%) | 1324(13.15%) | 47(0.47%) | 38(0.38%) | 155(1.54%) | 103(1.02%) | 302(3.00%) | 5781(57.41%) | 910(9.04%) | 363(3.61%) | 26(0.26%) | 79(0.78%) | 399(3.96%) | 18(0.18%) |  |
| 2002 | 9371 | 251(2.68%) | 1216(12.98%) | 36(0.38%) | 34(0.36%) | 154(1.64%) | 114(1.22%) | 325(3.47%) | 5385(57.46%) | 891(9.51%) | 325(3.47%) | 21(0.22%) | 70(0.75%) | 344(3.67%) | 17(0.18%) |  |
| 2003 | 8805 | 258(2.93%) | 1176(13.36%) | 37(0.42%) | 38(0.43%) | 139(1.58%) | 123(1.40%) | 327(3.71%) | 4989(56.66%) | 779(8.85%) | 294(3.34%) | 23(0.26%) | 70(0.80%) | 351(3.99%) | 10(0.11%) |  |
| 2004 | 8000 | 232(2.90%) | 1075(13.44%) | 36(0.45%) | 30(0.38%) | 138(1.73%) | 110(1.38%) | 313(3.91%) | 4431(55.39%) | 706(8.83%) | 297(3.71%) | 28(0.35%) | 71(0.89%) | 314(3.93%) | 14(0.18%) |  |
| 2005 | 7660 | 254(3.32%) | 1006(13.13%) | 34(0.44%) | 27(0.35%) | 142(1.85%) | 122(1.59%) | 301(3.93%) | 4209(54.95%) | 763(9.96%) | 271(3.54%) | 14(0.18%) | 58(0.76%) | 269(3.51%) | 9(0.12%) |  |
| 2006 | 7008 | 223(3.18%) | 989(14.11%) | 36(0.51%) | 25(0.36%) | 127(1.81%) | 207(2.95%) | 279(3.98%) | 3601(51.38%) | 652(9.30%) | 281(4.01%) | 21(0.30%) | 74(1.06%) | 289(4.12%) | 15(0.21%) |  |
| 2007 | 6272 | 186(2.97%) | 847(13.50%) | 25(0.40%) | 27(0.43%) | 143(2.28%) | 164(2.61%) | 266(4.24%) | 3263(52.02%) | 613(9.77%) | 226(3.60%) | 19(0.30%) | 40(0.64%) | 240(3.83%) | 9(0.14%) |  |
| 2008 | 6197 | 198(3.20%) | 835(13.47%) | 31(0.50%) | 25(0.40%) | 102(1.65%) | 202(3.26%) | 274(4.42%) | 3151(50.85%) | 670(10.81%) | 222(3.58%) | 23(0.37%) | 51(0.82%) | 219(3.53%) | 10(0.16%) |  |
| 2009 | 5771 | 209(3.62%) | 779(13.50%) | 29(0.50%) | 24(0.42%) | 131(2.27%) | 170(2.95%) | 240(4.16%) | 2951(51.13%) | 619(10.73%) | 209(3.62%) | 13(0.23%) | 51(0.88%) | 205(3.55%) | 9(0.16%) |  |
| 2010 | 5467 | 155(2.84%) | 761(13.92%) | 26(0.48%) | 20(0.37%) | 116(2.12%) | 181(3.31%) | 240(4.39%) | 2770(50.67%) | 558(10.21%) | 196(3.59%) | 14(0.26%) | 59(1.08%) | 180(3.29%) | 10(0.18%) |  |
| 2011 | 4961 | 156(3.14%) | 728(14.67%) | 20(0.40%) | 24(0.48%) | 116(2.34%) | 181(3.65%) | 242(4.88%) | 2490(50.19%) | 532(10.72%) | 187(3.77%) | 16(0.32%) | 42(0.85%) | 60(1.21%) | 10(0.20%) |  |
| 2012 | 4712 | 131(2.78%) | 669(14.20%) | 25(0.53%) | 21(0.45%) | 122(2.59%) | 228(4.84%) | 218(4.63%) | 2336(49.58%) | 496(10.53%) | 156(3.31%) | 16(0.34%) | 41(0.87%) | 59(1.25%) | 11(0.23%) |  |
| 2013 | 4453 | 157(3.53%) | 632(14.19%) | 18(0.40%) | 21(0.47%) | 119(2.67%) | 195(4.38%) | 229(5.14%) | 2191(49.20%) | 422(9.48%) | 168(3.77%) | 21(0.47%) | 45(1.01%) | 53(1.19%) | 12(0.27%) |  |
| 2014 | 4152 | 115(2.77%) | 529(12.74%) | 13(0.31%) | 18(0.43%) | 94(2.26%) | 191(4.60%) | 179(4.31%) | 2131(51.32%) | 433(10.43%) | 157(3.78%) | 8(0.19%) | 45(1.08%) | 54(1.30%) | 8(0.19%) |  |
| 2015 | 3850 | 111(2.88%) | 505(13.12%) | 16(0.42%) | 25(0.65%) | 105(2.73%) | 161(4.18%) | 214(5.56%) | 1883(48.91%) | 399(10.36%) | 159(4.13%) | 17(0.44%) | 46(1.19%) | 52(1.35%) | 6(0.16%) |  |
| 2016 | 3879 | 109(2.81%) | 516(13.30%) | 16(0.41%) | 16(0.41%) | 116(2.99%) | 132(3.40%) | 240(6.19%) | 1875(48.34%) | 410(10.57%) | 159(4.10%) | 16(0.41%) | 36(0.93%) | 48(1.24%) | 8(0.21%) |  |
| 2017 | 3982 | 135(3.39%) | 530(13.31%) | 16(0.40%) | 16(0.40%) | 125(3.14%) | 180(4.52%) | 219(5.50%) | 1892(47.51%) | 394(9.89%) | 168(4.22%) | 14(0.35%) | 39(0.98%) | 49(1.23%) | 15(0.38%) |  |
| 2018 | 3801 | 135(3.55%) | 486(12.79%) | 17(0.45%) | 20(0.53%) | 121(3.18%) | 141(3.71%) | 232(6.10%) | 1720(45.25%) | 421(11.08%) | 150(3.95%) | 18(0.47%) | 46(1.21%) | 58(1.53%) | 8(0.21%) |  |
| 2019 | 3607 | 116(3.22%) | 489(13.56%) | 24(0.67%) | 16(0.44%) | 100(2.77%) | 143(3.96%) | 229(6.35%) | 1642(45.52%) | 364(10.09%) | 171(4.74%) | 17(0.47%) | 38(1.05%) | 48(1.33%) | 15(0.42%) |  |
| 2020 | 4709 | 129(2.74%) | 537(11.40%) | 25(0.53%) | 21(0.45%) | 117(2.48%) | 182(3.86%) | 243(5.16%) | 1829(38.84%) | 378(8.03%) | 174(3.70%) | 16(0.34%) | 53(1.13%) | 55(1.17%) | 20(0.42%) | 670(14.23%) |
| 2021 | 4615 | 131(2.84%) | 494(10.70%) | 16(0.35%) | 22(0.48%) | 140(3.03%) | 154(3.34%) | 238(5.16%) | 1678(36.36%) | 290(6.28%) | 175(3.79%) | 16(0.35%) | 44(0.95%) | 71(1.54%) | 11(0.24%) | 876(18.98%) |
| 2022 | 3928 | 120(3.05%) | 497(12.65%) | 21(0.53%) | 18(0.46%) | 140(3.56%) | 154(3.92%) | 237(6.03%) | 1563(39.79%) | 257(6.54%) | 171(4.35%) | 16(0.41%) | 45(1.15%) | 58(1.48%) | 11(0.28%) | 340(8.66%) |
| 2023 | 3396 | 114(3.36%) | 457(13.46%) | 13(0.38%) | 23(0.68%) | 119(3.50%) | 145(4.27%) | 201(5.92%) | 1451(42.73%) | 271(7.98%) | 153(4.51%) | 17(0.50%) | 47(1.38%) | 39(1.15%) | 18(0.53%) | 83(2.44%) |
| 2024 | 3419 | 104(3.04%) | 448(13.10%) | 9(0.26%) | 18(0.53%) | 128(3.74%) | 157(4.59%) | 222(6.49%) | 1413(41.33%) | 290(8.48%) | 183(5.35%) | 14(0.41%) | 49(1.43%) | 40(1.17%) | 12(0.35%) | 51(1.49%) |
| Total | 155684 | 4687(3.01%) | 20607(13.24%) | 686(0.44%) | 662(0.43%) | 3320(2.13%) | 4082(2.62%) | 6667(4.28%) | 80753(51.87%) | 14563(9.35%) | 5772(3.71%) | 457(0.29%) | 1406(0.90%) | 4432(2.85%) | 316(0.20%) | 2020(1.30%) |

**Supplementary Table 6. Annual burden, recorded diabetes subtype composition, and underlying-cause distribution of E11-nonUCD, United States, 1999–2024.**

| **Year** | **nonUCD (n)** | **A00–B99 n(%)** | **C00–C97 n(%)** | **D00-D48 n(%)** | **D50-D89 n(%)** | **E00–E09, E15–E88 n(%)** | **F01–F99 n(%)** | **G00–G98 n(%)** | **I00–I99 n(%)** | **J00–J98 n(%)** | **K00–K92 n(%)** | **L00-L98 n(%)** | **M00-M99 n(%)** | **N00–N98 n(%)** | **Q00-Q99 n(%)** | **U07.1 n(%)** |
| --- | --- | --- | --- | --- | --- | --- | --- | --- | --- | --- | --- | --- | --- | --- | --- | --- |
| **E11** | | | | | | | | | | | | | | | | |
| 1999 | 27915 | 587(2.10%) | 4685(16.78%) | 139(0.50%) | 70(0.25%) | 403(1.44%) | 342(1.23%) | 988(3.54%) | 16012(57.36%) | 2522(9.03%) | 935(3.35%) | 26(0.09%) | 154(0.55%) | 702(2.51%) | 29(0.10%) |  |
| 2000 | 30521 | 589(1.93%) | 5140(16.84%) | 162(0.53%) | 101(0.33%) | 483(1.58%) | 430(1.41%) | 1292(4.23%) | 16957(55.56%) | 2852(9.34%) | 1055(3.46%) | 40(0.13%) | 204(0.67%) | 876(2.87%) | 34(0.11%) |  |
| 2001 | 32559 | 670(2.06%) | 5684(17.46%) | 175(0.54%) | 117(0.36%) | 522(1.60%) | 528(1.62%) | 1355(4.16%) | 17698(54.36%) | 3056(9.39%) | 1212(3.72%) | 39(0.12%) | 229(0.70%) | 895(2.75%) | 27(0.08%) |  |
| 2002 | 34969 | 871(2.49%) | 6015(17.20%) | 193(0.55%) | 136(0.39%) | 606(1.73%) | 617(1.76%) | 1603(4.58%) | 18452(52.77%) | 3473(9.93%) | 1275(3.65%) | 51(0.15%) | 270(0.77%) | 989(2.83%) | 31(0.09%) |  |
| 2003 | 36697 | 858(2.34%) | 6379(17.38%) | 188(0.51%) | 134(0.37%) | 676(1.84%) | 636(1.73%) | 1799(4.90%) | 18953(51.65%) | 3692(10.06%) | 1420(3.87%) | 67(0.18%) | 301(0.82%) | 1124(3.06%) | 34(0.09%) |  |
| 2004 | 37954 | 922(2.43%) | 6744(17.77%) | 196(0.52%) | 147(0.39%) | 713(1.88%) | 662(1.74%) | 1957(5.16%) | 19287(50.82%) | 3915(10.32%) | 1415(3.73%) | 61(0.16%) | 283(0.75%) | 1157(3.05%) | 38(0.10%) |  |
| 2005 | 41074 | 1066(2.60%) | 7216(17.57%) | 219(0.53%) | 158(0.38%) | 796(1.94%) | 960(2.34%) | 2301(5.60%) | 20197(49.17%) | 4412(10.74%) | 1587(3.86%) | 88(0.21%) | 290(0.71%) | 1251(3.05%) | 39(0.09%) |  |
| 2006 | 42180 | 1137(2.70%) | 7545(17.89%) | 264(0.63%) | 158(0.37%) | 836(1.98%) | 1618(3.84%) | 2450(5.81%) | 19863(47.09%) | 4385(10.40%) | 1627(3.86%) | 128(0.30%) | 321(0.76%) | 1321(3.13%) | 44(0.10%) |  |
| 2007 | 43625 | 1162(2.66%) | 7962(18.25%) | 254(0.58%) | 215(0.49%) | 853(1.96%) | 1632(3.74%) | 2691(6.17%) | 20053(45.97%) | 4657(10.68%) | 1663(3.81%) | 126(0.29%) | 349(0.80%) | 1414(3.24%) | 40(0.09%) |  |
| 2008 | 45607 | 1160(2.54%) | 8255(18.10%) | 309(0.68%) | 204(0.45%) | 909(1.99%) | 2078(4.56%) | 2875(6.30%) | 20400(44.73%) | 5288(11.59%) | 1744(3.82%) | 132(0.29%) | 340(0.75%) | 1268(2.78%) | 53(0.12%) |  |
| 2009 | 46358 | 1311(2.83%) | 8651(18.66%) | 261(0.56%) | 184(0.40%) | 969(2.09%) | 2152(4.64%) | 3004(6.48%) | 20526(44.28%) | 5313(11.46%) | 1697(3.66%) | 129(0.28%) | 346(0.75%) | 1184(2.55%) | 44(0.09%) |  |
| 2010 | 47620 | 1287(2.70%) | 9014(18.93%) | 264(0.55%) | 189(0.40%) | 1047(2.20%) | 2478(5.20%) | 3375(7.09%) | 20498(43.04%) | 5143(10.80%) | 1775(3.73%) | 114(0.24%) | 394(0.83%) | 1359(2.85%) | 53(0.11%) |  |
| 2011 | 46718 | 1271(2.72%) | 8671(18.56%) | 281(0.60%) | 202(0.43%) | 1075(2.30%) | 2671(5.72%) | 3365(7.20%) | 20092(43.01%) | 5378(11.51%) | 1798(3.85%) | 157(0.34%) | 435(0.93%) | 588(1.26%) | 57(0.12%) |  |
| 2012 | 48142 | 1330(2.76%) | 8818(18.32%) | 277(0.58%) | 237(0.49%) | 1136(2.36%) | 2948(6.12%) | 3498(7.27%) | 20718(43.04%) | 5512(11.45%) | 1874(3.89%) | 143(0.30%) | 402(0.84%) | 547(1.14%) | 39(0.08%) |  |
| 2013 | 50074 | 1405(2.81%) | 9241(18.45%) | 315(0.63%) | 228(0.46%) | 1103(2.20%) | 3166(6.32%) | 3588(7.17%) | 21415(42.77%) | 5769(11.52%) | 1984(3.96%) | 137(0.27%) | 320(0.64%) | 650(1.30%) | 65(0.13%) |  |
| 2014 | 49858 | 1314(2.64%) | 9207(18.47%) | 303(0.61%) | 236(0.47%) | 1212(2.43%) | 3039(6.10%) | 3751(7.52%) | 21536(43.19%) | 5470(10.97%) | 1954(3.92%) | 146(0.29%) | 336(0.67%) | 583(1.17%) | 37(0.07%) |  |
| 2015 | 54223 | 1447(2.67%) | 9774(18.03%) | 315(0.58%) | 226(0.42%) | 1328(2.45%) | 2969(5.48%) | 4251(7.84%) | 23477(43.30%) | 6093(11.24%) | 2174(4.01%) | 168(0.31%) | 424(0.78%) | 702(1.29%) | 70(0.13%) |  |
| 2016 | 63132 | 1604(2.54%) | 11888(18.83%) | 370(0.59%) | 266(0.42%) | 1616(2.56%) | 3319(5.26%) | 5175(8.20%) | 27043(42.84%) | 6717(10.64%) | 2557(4.05%) | 217(0.34%) | 488(0.77%) | 795(1.26%) | 71(0.11%) |  |
| 2017 | 70173 | 1663(2.37%) | 13020(18.55%) | 369(0.53%) | 294(0.42%) | 1840(2.62%) | 3743(5.33%) | 6088(8.68%) | 29805(42.47%) | 7812(11.13%) | 2675(3.81%) | 211(0.30%) | 498(0.71%) | 898(1.28%) | 70(0.10%) |  |
| 2018 | 74337 | 1665(2.24%) | 13790(18.55%) | 401(0.54%) | 301(0.40%) | 1990(2.68%) | 3794(5.10%) | 6519(8.77%) | 31629(42.55%) | 8231(11.07%) | 2915(3.92%) | 241(0.32%) | 540(0.73%) | 920(1.24%) | 80(0.11%) |  |
| 2019 | 78105 | 1655(2.12%) | 14468(18.52%) | 438(0.56%) | 314(0.40%) | 2190(2.80%) | 3925(5.03%) | 7093(9.08%) | 33035(42.30%) | 8401(10.76%) | 3056(3.91%) | 236(0.30%) | 602(0.77%) | 963(1.23%) | 77(0.10%) |  |
| 2020 | 115388 | 2041(1.77%) | 17164(14.88%) | 528(0.46%) | 404(0.35%) | 2814(2.44%) | 5029(4.36%) | 8969(7.77%) | 39434(34.18%) | 9620(8.34%) | 3959(3.43%) | 295(0.26%) | 758(0.66%) | 1180(1.02%) | 85(0.07%) | 21177(18.35%) |
| 2021 | 123557 | 2135(1.73%) | 18292(14.80%) | 535(0.43%) | 413(0.33%) | 3305(2.67%) | 4901(3.97%) | 8834(7.15%) | 41943(33.95%) | 9318(7.54%) | 4287(3.47%) | 322(0.26%) | 880(0.71%) | 1376(1.11%) | 132(0.11%) | 24471(19.81%) |
| 2022 | 114483 | 2255(1.97%) | 18825(16.44%) | 556(0.49%) | 446(0.39%) | 3568(3.12%) | 5199(4.54%) | 9705(8.48%) | 44378(38.76%) | 9885(8.63%) | 4553(3.98%) | 364(0.32%) | 946(0.83%) | 1366(1.19%) | 135(0.12%) | 9730(8.50%) |
| 2023 | 107038 | 2363(2.21%) | 19527(18.24%) | 564(0.53%) | 453(0.42%) | 3425(3.20%) | 5165(4.83%) | 10022(9.36%) | 43365(40.51%) | 9741(9.10%) | 4528(4.23%) | 379(0.35%) | 921(0.86%) | 1354(1.26%) | 161(0.15%) | 2490(2.33%) |
| 2024 | 110757 | 2364(2.13%) | 20756(18.74%) | 562(0.51%) | 477(0.43%) | 3684(3.33%) | 5182(4.68%) | 10631(9.60%) | 45361(40.96%) | 9899(8.94%) | 4671(4.22%) | 352(0.32%) | 1003(0.91%) | 1279(1.15%) | 144(0.13%) | 1542(1.39%) |
| Total | 1573064 | 36132(2.30%) | 276731(17.59%) | 8438(0.54%) | 6310(0.40%) | 39099(2.49%) | 69183(4.40%) | 117179(7.45%) | 672127(42.73%) | 156554(9.95%) | 60390(3.84%) | 4369(0.28%) | 12034(0.77%) | 26741(1.70%) | 1689(0.11%) | 59410(3.78%) |

**Supplementary Table 7. Period-specific underlying-cause distribution of E10-nonUCD deaths, United States, 1999–2024.**

| Condition | 1999-2004 | 2005-2009 | 2010-2014 | 2015-2019 | 2020-2024 | Total |
| --- | --- | --- | --- | --- | --- | --- |
| A00–B99 | 1699(2.84%) | 1070(3.25%) | 714(3.01%) | 606(3.17%) | 598(2.98%) | 4687(3.01%) |
| C00–C97 | 7873(13.16%) | 4456(13.54%) | 3319(13.98%) | 2526(13.21%) | 2433(12.12%) | 20607(13.24%) |
| D00-D48 | 256(0.43%) | 155(0.47%) | 102(0.43%) | 89(0.47%) | 84(0.42%) | 686(0.44%) |
| D50-D89 | 235(0.39%) | 128(0.39%) | 104(0.44%) | 93(0.49%) | 102(0.51%) | 662(0.43%) |
| E00–E09, E15–E88 | 897(1.50%) | 645(1.96%) | 567(2.39%) | 567(2.97%) | 644(3.21%) | 3320(2.13%) |
| F01–F99 | 692(1.16%) | 865(2.63%) | 976(4.11%) | 757(3.96%) | 792(3.95%) | 4082(2.62%) |
| G00–G98 | 1924(3.21%) | 1360(4.13%) | 1108(4.67%) | 1134(5.93%) | 1141(5.69%) | 6667(4.28%) |
| I00–I99 | 34714(58.01%) | 17175(52.19%) | 11918(50.19%) | 9012(47.14%) | 7934(39.54%) | 80753(51.87%) |
| J00–J98 | 5331(8.91%) | 3317(10.08%) | 2441(10.28%) | 1988(10.40%) | 1486(7.41%) | 14563(9.35%) |
| K00–K92 | 2036(3.40%) | 1209(3.67%) | 864(3.64%) | 807(4.22%) | 856(4.27%) | 5772(3.71%) |
| L00-L98 | 131(0.22%) | 90(0.27%) | 75(0.32%) | 82(0.43%) | 79(0.39%) | 457(0.29%) |
| M00-M99 | 457(0.76%) | 274(0.83%) | 232(0.98%) | 205(1.07%) | 238(1.19%) | 1406(0.90%) |
| N00–N98 | 2286(3.82%) | 1222(3.71%) | 406(1.71%) | 255(1.33%) | 263(1.31%) | 4432(2.85%) |
| Q00-Q99 | 89(0.15%) | 52(0.16%) | 51(0.21%) | 52(0.27%) | 72(0.36%) | 316(0.20%) |
| U07.1 | 0(0.00%) | 0(0.00%) | 0(0.00%) | 0(0.00%) | 2020(10.07%) | 2020(1.30%) |
| Others | 1225(2.05%) | 890(2.70%) | 868(3.66%) | 946(4.95%) | 1325(6.60%) | 5254(3.37%) |
| NonUCD | 59845 | 32908 | 23745 | 19119 | 20067 | 155684 |

**Others:** Others was calculated as the corresponding nonUCD total minus the sum of deaths assigned to A00–B99, C00–C97, D00–D48, D50–D89, E00–E09 and E15–E88, F01–F99, G00–G98, I00–I99, J00–J98, K00–K92, L00–L98, M00–M99, N00–N98, Q00–Q99, and U07.1 as the underlying cause of death.

**Supplementary Table 8. Distribution of underlying causes among deaths with diabetes (E10–E14) recorded as a non-underlying cause, by age group and study period, 1999–2024.**

| Underlying cause | 1999-2004 | 2005-2009 | 2010-2014 | 2015-2019 | 2020-2024 | Total |
| --- | --- | --- | --- | --- | --- | --- |
|  |  | Age <65 years (E10-E14) | | |  |  |
| A00-B99 | 8019(4.48%) | 8176(4.64%) | 8300(4.43%) | 7533(3.75%) | 8026(2.81%) | 40054(3.89%) |
| C00-C97 | 24441(13.65%) | 25805(14.63%) | 28058(14.98%) | 28502(14.21%) | 34869(12.22%) | 141675(13.77%) |
| D00-D48 | 588(0.33%) | 529(0.30%) | 561(0.30%) | 604(0.30%) | 654(0.23%) | 2936(0.29%) |
| D50-D89 | 971(0.54%) | 937(0.53%) | 947(0.51%) | 980(0.49%) | 1242(0.44%) | 5077(0.49%) |
| E00-E09, E15-E88 | 4564(2.55%) | 5539(3.14%) | 6346(3.39%) | 7183(3.58%) | 9060(3.17%) | 32692(3.18%) |
| F01-F99 | 1599(0.89%) | 1845(1.05%) | 2565(1.37%) | 3349(1.67%) | 5063(1.77%) | 14421(1.40%) |
| G00-G98 | 3326(1.86%) | 3896(2.21%) | 4365(2.33%) | 4810(2.40%) | 6004(2.10%) | 22401(2.18%) |
| I00-I99 | 101212(56.54%) | 91510(51.88%) | 96089(51.31%) | 102768(51.22%) | 120753(42.31%) | 512332(49.80%) |
| J00-J98 | 12349(6.90%) | 14321(8.12%) | 16083(8.59%) | 17780(8.86%) | 19218(6.73%) | 79751(7.75%) |
| K00-K92 | 8991(5.02%) | 9303(5.27%) | 10088(5.39%) | 10915(5.44%) | 15075(5.28%) | 54372(5.29%) |
| L00-L98 | 527(0.29%) | 727(0.41%) | 836(0.45%) | 922(0.46%) | 1263(0.44%) | 4275(0.42%) |
| M00-M99 | 1706(0.95%) | 1788(1.01%) | 1774(0.95%) | 1783(0.89%) | 2558(0.90%) | 9609(0.93%) |
| N00-N98 | 5455(3.05%) | 5109(2.90%) | 2419(1.29%) | 2026(1.01%) | 2831(0.99%) | 17840(1.73%) |
| Q00-Q99 | 420(0.23%) | 419(0.24%) | 512(0.27%) | 582(0.29%) | 787(0.28%) | 2720(0.26%) |
| U07.1 | 0(0.00%) | 0(0.00%) | 0(0.00%) | 0(0.00%) | 41325(14.48%) | 41325(4.02%) |
|  |  | Age ≥65 years(E10-E14) | | |  |  |
| A00-B99 | 15577(2.20%) | 14929(2.39%) | 15245(2.35%) | 15650(2.16%) | 20543(1.95%) | 81944(2.18%) |
| C00-C97 | 105267(14.84%) | 99921(15.96%) | 106764(16.43%) | 116449(16.07%) | 155508(14.73%) | 583909(15.51%) |
| D00-D48 | 3581(0.50%) | 3590(0.57%) | 3750(0.58%) | 3933(0.54%) | 4872(0.46%) | 19726(0.52%) |
| D50-D89 | 2222(0.31%) | 2174(0.35%) | 2464(0.38%) | 2714(0.37%) | 3513(0.33%) | 13087(0.35%) |
| E00-E09, E15-E88 | 8213(1.16%) | 8350(1.33%) | 10191(1.57%) | 14085(1.94%) | 24656(2.34%) | 65495(1.74%) |
| F01-F99 | 10505(1.48%) | 25639(4.10%) | 42828(6.59%) | 42028(5.80%) | 50337(4.77%) | 171337(4.55%) |
| G00-G98 | 25843(3.64%) | 32942(5.26%) | 41004(6.31%) | 56082(7.74%) | 81455(7.72%) | 237326(6.30%) |
| I00-I99 | 407985(57.51%) | 312535(49.93%) | 302874(46.60%) | 332728(45.91%) | 426266(40.39%) | 1782388(47.34%) |
| J00-J98 | 67871(9.57%) | 66875(10.68%) | 70973(10.92%) | 76929(10.62%) | 87426(8.28%) | 370074(9.83%) |
| K00-K92 | 23083(3.25%) | 20327(3.25%) | 20359(3.13%) | 23936(3.30%) | 34298(3.25%) | 122003(3.24%) |
| L00-L98 | 1053(0.15%) | 1631(0.26%) | 1736(0.27%) | 2053(0.28%) | 3108(0.29%) | 9581(0.25%) |
| M00-M99 | 4584(0.65%) | 4254(0.68%) | 4313(0.66%) | 4744(0.65%) | 7451(0.71%) | 25346(0.67%) |
| N00-N98 | 22752(3.21%) | 19695(3.15%) | 10167(1.56%) | 9367(1.29%) | 12838(1.22%) | 74819(1.99%) |
| Q00-Q99 | 457(0.06%) | 343(0.05%) | 372(0.06%) | 428(0.06%) | 654(0.06%) | 2254(0.06%) |
| U07.1 | 0(0.00%) | 0(0.00%) | 0(0.00%) | 0(0.00%) | 105844(10.03%) | 105844(2.81%) |

**Supplementary Table 9. Distribution of underlying causes among deaths with diabetes (E11) recorded as a non-underlying cause, by age group and study period, 1999–2024.**

| Underlying cause | 1999-2004 | 2005-2009 | 2010-2014 | 2015-2019 | 2020-2024 | Total |
| --- | --- | --- | --- | --- | --- | --- |
|  |  | Age <65 years(E11) | | |  |  |
| A00-B99 | 1242(4.34%) | 1829(5.11%) | 2096(5.20%) | 2322(4.25%) | 2735(2.85%) | 10224(4.01%) |
| C00-C97 | 5719(19.98%) | 7407(20.71%) | 8475(21.01%) | 11373(20.80%) | 16252(16.96%) | 49226(19.28%) |
| D00-D48 | 112(0.39%) | 129(0.36%) | 159(0.39%) | 230(0.42%) | 271(0.28%) | 901(0.35%) |
| D50-D89 | 166(0.58%) | 228(0.64%) | 237(0.59%) | 307(0.56%) | 509(0.53%) | 1447(0.57%) |
| E00-E09, E15-E88 | 959(3.35%) | 1398(3.91%) | 1758(4.36%) | 2459(4.50%) | 3682(3.84%) | 10256(4.02%) |
| F01-F99 | 197(0.69%) | 333(0.93%) | 498(1.23%) | 730(1.33%) | 1402(1.46%) | 3160(1.24%) |
| G00-G98 | 644(2.25%) | 955(2.67%) | 1168(2.90%) | 1657(3.03%) | 2446(2.55%) | 6870(2.69%) |
| I00-I99 | 14147(49.43%) | 15543(43.46%) | 16881(41.84%) | 23230(42.48%) | 34415(35.91%) | 104216(40.83%) |
| J00-J98 | 2355(8.23%) | 3538(9.89%) | 4224(10.47%) | 5686(10.40%) | 7338(7.66%) | 23141(9.07%) |
| K00-K92 | 1553(5.43%) | 2175(6.08%) | 2695(6.68%) | 3623(6.62%) | 6121(6.39%) | 16167(6.33%) |
| L00-L98 | 65(0.23%) | 155(0.43%) | 197(0.49%) | 270(0.49%) | 442(0.46%) | 1129(0.44%) |
| M00-M99 | 297(1.04%) | 380(1.06%) | 479(1.19%) | 584(1.07%) | 1008(1.05%) | 2748(1.08%) |
| N00-N98 | 683(2.39%) | 996(2.79%) | 568(1.41%) | 621(1.14%) | 1007(1.05%) | 3875(1.52%) |
| Q00-Q99 | 74(0.26%) | 93(0.26%) | 115(0.29%) | 188(0.34%) | 335(0.35%) | 805(0.32%) |
| U07.1 | 0(0.00%) | 0(0.00%) | 0(0.00%) | 0(0.00%) | 14880(15.52%) | 14880(5.83%) |
|  | Age ³65 years(E11) | | | | | |
| A00-B99 | 3255(1.89%) | 4007(2.19%) | 4511(2.23%) | 5710(2.00%) | 8423(1.77%) | 25906(1.97%) |
| C00-C97 | 28926(16.82%) | 32222(17.60%) | 36476(18.05%) | 51567(18.08%) | 78311(16.47%) | 227502(17.26%) |
| D00-D48 | 941(0.55%) | 1178(0.64%) | 1281(0.63%) | 1663(0.58%) | 2474(0.52%) | 7537(0.57%) |
| D50-D89 | 539(0.31%) | 691(0.38%) | 855(0.42%) | 1094(0.38%) | 1684(0.35%) | 4863(0.37%) |
| E00-E09, E15-E88 | 2444(1.42%) | 2965(1.62%) | 3815(1.89%) | 6505(2.28%) | 13114(2.76%) | 28843(2.19%) |
| F01-F99 | 3018(1.75%) | 8107(4.43%) | 13804(6.83%) | 17020(5.97%) | 24074(5.06%) | 66023(5.01%) |
| G00-G98 | 8350(4.85%) | 12366(6.75%) | 16408(8.12%) | 27469(9.63%) | 45715(9.62%) | 110308(8.37%) |
| I00-I99 | 93211(54.20%) | 85493(46.70%) | 87377(43.24%) | 121755(42.68%) | 180063(37.88%) | 567899(43.10%) |
| J00-J98 | 17155(9.97%) | 20517(11.21%) | 23048(11.41%) | 31568(11.07%) | 41124(8.65%) | 133412(10.12%) |
| K00-K92 | 5759(3.35%) | 6143(3.36%) | 6690(3.31%) | 9753(3.42%) | 15877(3.34%) | 44222(3.36%) |
| L00-L98 | 219(0.13%) | 448(0.24%) | 500(0.25%) | 803(0.28%) | 1270(0.27%) | 3240(0.25%) |
| M00-M99 | 1144(0.67%) | 1266(0.69%) | 1408(0.70%) | 1968(0.69%) | 3499(0.74%) | 9285(0.70%) |
| N00-N98 | 5060(2.94%) | 5442(2.97%) | 3159(1.56%) | 3657(1.28%) | 5548(1.17%) | 22866(1.74%) |
| Q00-Q99 | 119(0.07%) | 127(0.07%) | 136(0.07%) | 180(0.06%) | 322(0.07%) | 884(0.07%) |
| U07.1 | 0(0.00%) | 0(0.00%) | 0(0.00%) | 0(0.00%) | 44529(9.37%) | 44529(3.38%) |

**Supplementary Table 10. Distribution of underlying causes among deaths with diabetes (E10) recorded as a non-underlying cause, by age group and study period, 1999–2024.**

| Underlying cause | 1999-2004 | 2005-2009 | 2010-2014 | 2015-2019 | 2020-2024 | Total |
| --- | --- | --- | --- | --- | --- | --- |
|  |  | Age <65 years(E10) | | |  |  |
| A00-B99 | 691(4.54%) | 493(4.95%) | 358(4.54%) | 325(4.72%) | 331(4.12%) | 2198(4.58%) |
| C00-C97 | 1747(11.48%) | 1166(11.70%) | 942(11.94%) | 744(10.80%) | 783(9.75%) | 5382(11.21%) |
| D00-D48 | 44(0.29%) | 24(0.24%) | 13(0.16%) | 25(0.36%) | 23(0.29%) | 129(0.27%) |
| D50-D89 | 95(0.62%) | 48(0.48%) | 44(0.56%) | 49(0.71%) | 52(0.65%) | 288(0.60%) |
| E00-E09, E15-E88 | 358(2.35%) | 318(3.19%) | 281(3.56%) | 280(4.06%) | 314(3.91%) | 1551(3.23%) |
| F01-F99 | 148(0.97%) | 109(1.09%) | 125(1.58%) | 143(2.08%) | 187(2.33%) | 712(1.48%) |
| G00-G98 | 350(2.30%) | 284(2.85%) | 238(3.02%) | 269(3.90%) | 290(3.61%) | 1431(2.98%) |
| I00-I99 | 8511(55.92%) | 5102(51.18%) | 3975(50.40%) | 3179(46.13%) | 3002(37.37%) | 23769(49.52%) |
| J00-J98 | 1117(7.34%) | 860(8.63%) | 667(8.46%) | 631(9.16%) | 528(6.57%) | 3803(7.92%) |
| K00-K92 | 744(4.89%) | 511(5.13%) | 417(5.29%) | 419(6.08%) | 476(5.92%) | 2567(5.35%) |
| L00-L98 | 63(0.41%) | 24(0.24%) | 36(0.46%) | 38(0.55%) | 45(0.56%) | 206(0.43%) |
| M00-M99 | 147(0.97%) | 118(1.18%) | 108(1.37%) | 98(1.42%) | 121(1.51%) | 592(1.23%) |
| N00-N98 | 621(4.08%) | 385(3.86%) | 126(1.60%) | 85(1.23%) | 104(1.29%) | 1321(2.75%) |
| Q00-Q99 | 47(0.31%) | 42(0.42%) | 40(0.51%) | 37(0.54%) | 53(0.66%) | 219(0.46%) |
| U07.1 | 0(0.00%) | 0(0.00%) | 0(0.00%) | 0(0.00%) | 920(11.45%) | 920(1.92%) |
|  | Age ³65 years(E10) | | | | | |
| A00-B99 | 1008(2.26%) | 577(2.52%) | 356(2.24%) | 281(2.30%) | 267(2.22%) | 2489(2.31%) |
| C00-C97 | 6126(13.73%) | 3290(14.34%) | 2377(14.99%) | 1782(14.57%) | 1666(13.85%) | 15241(14.15%) |
| D00-D48 | 212(0.48%) | 131(0.57%) | 89(0.56%) | 64(0.52%) | 61(0.51%) | 557(0.52%) |
| D50-D89 | 140(0.31%) | 80(0.35%) | 60(0.38%) | 44(0.36%) | 50(0.42%) | 374(0.35%) |
| E00-E09, E15-E88 | 539(1.21%) | 327(1.43%) | 286(1.80%) | 287(2.35%) | 330(2.74%) | 1769(1.64%) |
| F01-F99 | 544(1.22%) | 756(3.30%) | 851(5.37%) | 614(5.02%) | 605(5.03%) | 3370(3.13%) |
| G00-G98 | 1574(3.53%) | 1076(4.69%) | 870(5.49%) | 865(7.07%) | 851(7.07%) | 5236(4.86%) |
| I00-I99 | 26203(58.72%) | 12072(52.63%) | 7943(50.09%) | 5832(47.70%) | 4932(40.99%) | 56982(52.92%) |
| J00-J98 | 4214(9.44%) | 2457(10.71%) | 1774(11.19%) | 1357(11.10%) | 958(7.96%) | 10760(9.99%) |
| K00-K92 | 1292(2.90%) | 698(3.04%) | 447(2.82%) | 388(3.17%) | 380(3.16%) | 3205(2.98%) |
| L00-L98 | 72(0.16%) | 62(0.27%) | 39(0.25%) | 44(0.36%) | 34(0.28%) | 251(0.23%) |
| M00-M99 | 310(0.69%) | 156(0.68%) | 124(0.78%) | 107(0.88%) | 117(0.97%) | 814(0.76%) |
| N00-N98 | 1665(3.73%) | 837(3.65%) | 280(1.77%) | 170(1.39%) | 159(1.32%) | 3111(2.89%) |
| Q00-Q99 | 42(0.09%) | 10(0.04%) | 11(0.07%) | 15(0.12%) | 18(0.15%) | 96(0.09%) |
| U07.1 | 0(0.00%) | 0(0.00%) | 0(0.00%) | 0(0.00%) | 1100(9.14%) | 1100(1.02%) |

**Supplementary Table 11. Distribution of selected circulatory disease subtypes among DM-nonUCD deaths, United States, 1999–2024.**

| Year | nonUCD n | I10–I15 n(%) | I20–I25 n(%) | I70–I79 n(%) | I50 n(%) | I60–I69 n(%) |
| --- | --- | --- | --- | --- | --- | --- |
| **DM (E10-14)** | | | | | | |
| 1999 | 141371 | 5169(3.66%) | 55179(39.03%) | 833(0.59%) | 9042(6.40%) | 13278(9.39%) |
| 2000 | 143763 | 5576(3.88%) | 54398(37.84%) | 868(0.60%) | 9073(6.31%) | 12962(9.02%) |
| 2001 | 146755 | 5918(4.03%) | 54041(36.82%) | 913(0.62%) | 9332(6.36%) | 12945(8.82%) |
| 2002 | 150843 | 6433(4.26%) | 53436(35.42%) | 977(0.65%) | 9577(6.35%) | 13102(8.69%) |
| 2003 | 153357 | 7007(4.57%) | 52635(34.32%) | 1020(0.67%) | 9827(6.41%) | 12551(8.18%) |
| 2004 | 152313 | 7252(4.76%) | 50509(33.16%) | 1003(0.66%) | 10012(6.57%) | 12293(8.07%) |
| 2005 | 158499 | 7612(4.80%) | 51064(32.22%) | 1039(0.66%) | 10397(6.56%) | 11686(7.37%) |
| 2006 | 158588 | 7641(4.82%) | 49184(31.01%) | 974(0.61%) | 10432(6.58%) | 11118(7.01%) |
| 2007 | 160020 | 8028(5.02%) | 48116(30.07%) | 982(0.61%) | 10757(6.72%) | 10773(6.73%) |
| 2008 | 163276 | 8626(5.28%) | 47809(29.28%) | 964(0.59%) | 10515(6.44%) | 10973(6.72%) |
| 2009 | 161937 | 8881(5.48%) | 46821(28.91%) | 1036(0.64%) | 10523(6.50%) | 10147(6.27%) |
| 2010 | 164980 | 9011(5.46%) | 46564(28.22%) | 1087(0.66%) | 10642(6.45%) | 10374(6.29%) |
| 2011 | 165358 | 8427(5.10%) | 46695(28.24%) | 1104(0.67%) | 10720(6.48%) | 10451(6.32%) |
| 2012 | 167175 | 8828(5.28%) | 46812(28.00%) | 1148(0.69%) | 11011(6.59%) | 10472(6.26%) |
| 2013 | 171226 | 9495(5.55%) | 47360(27.66%) | 1229(0.72%) | 11384(6.65%) | 10488(6.13%) |
| 2014 | 168528 | 9619(5.71%) | 46301(27.47%) | 1240(0.74%) | 11559(6.86%) | 10104(6.00%) |
| 2015 | 173271 | 10522(6.07%) | 46536(26.86%) | 1293(0.75%) | 12235(7.06%) | 10509(6.07%) |
| 2016 | 178794 | 11328(6.34%) | 47291(26.45%) | 1341(0.75%) | 12544(7.02%) | 10943(6.12%) |
| 2017 | 187138 | 12297(6.57%) | 48544(25.94%) | 1370(0.73%) | 13336(7.13%) | 11471(6.13%) |
| 2018 | 190983 | 12759(6.68%) | 48913(25.61%) | 1331(0.70%) | 13399(7.02%) | 11473(6.01%) |
| 2019 | 195154 | 13653(7.00%) | 48956(25.09%) | 1492(0.76%) | 13627(6.98%) | 11732(6.01%) |
| 2020 | 285878 | 17045(5.96%) | 56882(19.90%) | 1748(0.61%) | 15546(5.44%) | 13866(4.85%) |
| 2021 | 296107 | 18224(6.15%) | 57042(19.26%) | 1949(0.66%) | 16327(5.51%) | 14874(5.02%) |
| 2022 | 268244 | 19060(7.11%) | 57209(21.33%) | 1857(0.69%) | 17136(6.39%) | 15507(5.78%) |
| 2023 | 244152 | 18862(7.73%) | 54437(22.30%) | 1737(0.71%) | 16730(6.85%) | 14870(6.09%) |
| 2024 | 246426 | 18909(7.67%) | 54729(22.21%) | 1817(0.74%) | 16982(6.89%) | 15522(6.30%) |
| **Total** | 4794136 | 276182(5.76%) | 1317463(27.48%) | 32352(0.67%) | 312665(6.52%) | 314484(6.56%) |
| **E10** | | | | | | |
| 1999 | 12438 | 401(3.22%) | 5021(40.37%) | 72(0.58%) | 749(6.02%) | 1098(8.83%) |
| 2000 | 11162 | 364(3.26%) | 4401(39.43%) | 71(0.64%) | 643(5.76%) | 996(8.92%) |
| 2001 | 10069 | 360(3.58%) | 3823(37.97%) | 60(0.60%) | 606(6.02%) | 791(7.86%) |
| 2002 | 9371 | 343(3.66%) | 3527(37.64%) | 58(0.62%) | 594(6.34%) | 762(8.13%) |
| 2003 | 8805 | 349(3.96%) | 3237(36.76%) | 60(0.68%) | 523(5.94%) | 725(8.23%) |
| 2004 | 8000 | 308(3.85%) | 2898(36.23%) | 58(0.73%) | 518(6.48%) | 568(7.10%) |
| 2005 | 7660 | 310(4.05%) | 2711(35.39%) | 45(0.59%) | 500(6.53%) | 552(7.21%) |
| 2006 | 7008 | 277(3.95%) | 2396(34.19%) | 38(0.54%) | 408(5.82%) | 422(6.02%) |
| 2007 | 6272 | 268(4.27%) | 2121(33.82%) | 39(0.62%) | 409(6.52%) | 371(5.92%) |
| 2008 | 6197 | 277(4.47%) | 2047(33.03%) | 45(0.73%) | 391(6.31%) | 352(5.68%) |
| 2009 | 5771 | 269(4.66%) | 1956(33.89%) | 42(0.73%) | 329(5.70%) | 313(5.42%) |
| 2010 | 5467 | 237(4.34%) | 1792(32.78%) | 40(0.73%) | 341(6.24%) | 301(5.51%) |
| 2011 | 4961 | 238(4.80%) | 1622(32.70%) | 36(0.73%) | 296(5.97%) | 260(5.24%) |
| 2012 | 4712 | 197(4.18%) | 1530(32.47%) | 28(0.59%) | 298(6.32%) | 242(5.14%) |
| 2013 | 4453 | 202(4.54%) | 1430(32.11%) | 39(0.88%) | 274(6.15%) | 218(4.90%) |
| 2014 | 4152 | 181(4.36%) | 1388(33.43%) | 22(0.53%) | 282(6.79%) | 227(5.47%) |
| 2015 | 3850 | 169(4.39%) | 1196(31.06%) | 44(1.14%) | 275(7.14%) | 173(4.49%) |
| 2016 | 3879 | 195(5.03%) | 1161(29.93%) | 34(0.88%) | 273(7.04%) | 187(4.82%) |
| 2017 | 3982 | 177(4.45%) | 1162(29.18%) | 34(0.85%) | 274(6.88%) | 211(5.30%) |
| 2018 | 3801 | 157(4.13%) | 1068(28.10%) | 32(0.84%) | 253(6.66%) | 182(4.79%) |
| 2019 | 3607 | 176(4.88%) | 975(27.03%) | 33(0.91%) | 258(7.15%) | 173(4.80%) |
| 2020 | 4709 | 203(4.31%) | 1098(23.32%) | 44(0.93%) | 251(5.33%) | 198(4.20%) |
| 2021 | 4615 | 182(3.94%) | 1008(21.84%) | 24(0.52%) | 232(5.03%) | 202(4.38%) |
| 2022 | 3928 | 181(4.61%) | 905(23.04%) | 39(0.99%) | 225(5.73%) | 183(4.66%) |
| 2023 | 3396 | 176(5.18%) | 830(24.44%) | 20(0.59%) | 225(6.63%) | 177(5.21%) |
| 2024 | 3419 | 161(4.71%) | 814(23.81%) | 27(0.79%) | 216(6.32%) | 158(4.62%) |
| **Total** | 155684 | 6358(4.08%) | 52117(33.48%) | 1084(0.70%) | 9643(6.19%) | 10042(6.45%) |
| **E11** | | | | | | |
| 1999 | 27915 | 957(3.43%) | 9983(35.76%) | 178(0.64%) | 1687(6.04%) | 2815(10.08%) |
| 2000 | 30521 | 1078(3.53%) | 10560(34.60%) | 171(0.56%) | 1856(6.08%) | 2869(9.40%) |
| 2001 | 32559 | 1148(3.53%) | 10914(33.52%) | 203(0.62%) | 2026(6.22%) | 2984(9.16%) |
| 2002 | 34969 | 1148(3.28%) | 11268(32.22%) | 237(0.68%) | 2139(6.12%) | 3223(9.22%) |
| 2003 | 36697 | 1409(3.84%) | 11429(31.14%) | 264(0.72%) | 2181(5.94%) | 3194(8.70%) |
| 2004 | 37954 | 1503(3.96%) | 11499(30.30%) | 239(0.63%) | 2349(6.19%) | 3230(8.51%) |
| 2005 | 41074 | 1638(3.99%) | 12008(29.24%) | 265(0.65%) | 2631(6.41%) | 3151(7.67%) |
| 2006 | 42180 | 1614(3.83%) | 11717(27.78%) | 280(0.66%) | 2740(6.50%) | 3125(7.41%) |
| 2007 | 43625 | 1691(3.88%) | 11802(27.05%) | 243(0.56%) | 2819(6.46%) | 3089(7.08%) |
| 2008 | 45607 | 1820(3.99%) | 11830(25.94%) | 285(0.62%) | 2850(6.25%) | 3221(7.06%) |
| 2009 | 46358 | 1824(3.93%) | 12022(25.93%) | 295(0.64%) | 2830(6.10%) | 3187(6.87%) |
| 2010 | 47620 | 1838(3.86%) | 11947(25.09%) | 321(0.67%) | 2877(6.04%) | 3137(6.59%) |
| 2011 | 46718 | 1674(3.58%) | 11632(24.90%) | 340(0.73%) | 2965(6.35%) | 3112(6.66%) |
| 2012 | 48142 | 1791(3.72%) | 11908(24.74%) | 361(0.75%) | 3085(6.41%) | 3195(6.64%) |
| 2013 | 50074 | 1983(3.96%) | 12191(24.35%) | 382(0.76%) | 3184(6.36%) | 3291(6.57%) |
| 2014 | 49858 | 1995(4.00%) | 12125(24.32%) | 396(0.79%) | 3394(6.81%) | 3208(6.43%) |
| 2015 | 54223 | 2331(4.30%) | 12941(23.87%) | 407(0.75%) | 3897(7.19%) | 3501(6.46%) |
| 2016 | 63132 | 2788(4.42%) | 14810(23.46%) | 505(0.80%) | 4285(6.79%) | 4196(6.65%) |
| 2017 | 70173 | 3326(4.74%) | 15913(22.68%) | 532(0.76%) | 4916(7.01%) | 4619(6.58%) |
| 2018 | 74337 | 3710(4.99%) | 16779(22.57%) | 530(0.71%) | 5250(7.06%) | 4868(6.55%) |
| 2019 | 78105 | 4233(5.42%) | 17264(22.10%) | 645(0.83%) | 5324(6.82%) | 5036(6.45%) |
| 2020 | 115388 | 5167(4.48%) | 20378(17.66%) | 748(0.65%) | 6266(5.43%) | 6279(5.44%) |
| 2021 | 123557 | 5950(4.82%) | 21257(17.20%) | 864(0.70%) | 6587(5.33%) | 6591(5.33%) |
| 2022 | 114483 | 6730(5.88%) | 21806(19.05%) | 843(0.74%) | 7150(6.25%) | 7121(6.22%) |
| 2023 | 107038 | 7027(6.56%) | 20978(19.60%) | 799(0.75%) | 6977(6.52%) | 6861(6.41%) |
| 2024 | 110757 | 7145(6.45%) | 21785(19.67%) | 888(0.80%) | 7318(6.61%) | 7505(6.78%) |
| **Total** | 1573064 | 73518(4.67%) | 368746(23.44%) | 11221(0.71%) | 99583(6.33%) | 106608(6.78%) |

**Supplementary Figure 1. Annual burden and recorded diabetes subtype composition of DM-nonUCD deaths, United States, 1999–2024.**

Annual death counts and proportions for E10-nonUCD and E11-nonUCD among all DM-nonUCD deaths. Percentages indicate the proportion of all DM-nonUCD deaths in each calendar year. Diabetes subtype analyses were based on ICD-10 codes recorded among the multiple causes of death.

DM, diabetes mellitus; DM-nonUCD, diabetes recorded as a non-underlying cause of death; E10-nonUCD, type 1 diabetes recorded as a non-underlying cause of death; E11-nonUCD, type 2 diabetes recorded as a non-underlying cause of death; ICD-10, International Classification of Diseases, 10th Revision.

**Supplementary Figure 2. Age-adjusted mortality trends for selected underlying causes among E10-nonUCD deaths, United States, 1999–2024.**

Annual age-adjusted mortality rates (AAMR) for underlying causes assigned to (A) circulatory diseases, (B) malignant neoplasms, (C) respiratory diseases, (D) infectious and parasitic diseases, (E) diseases of the nervous system, and (F) digestive diseases. Joinpoint segment bars below each plot indicate annual percentage changes (APC) for each fitted segment. Average annual percentage changes (AAPC) summarize the overall trend across the study period. Rates are expressed per 100,000 population and age-adjusted to the 2000 U.S. standard population. E10-nonUCD, type 1 diabetes recorded as a non-underlying cause of death.

**Supplementary Figure 3. Pre-pandemic age-adjusted mortality trends for selected underlying causes among DM-nonUCD deaths in the United States, 1999–2019.**

Annual age-adjusted mortality rates (AAMRs) are shown for deaths with the following underlying causes: (A) circulatory diseases, (B) malignant neoplasms, (C) diseases of the nervous system, (D) respiratory diseases, (E) mental and behavioral disorders, and (F) digestive diseases. Overall and sex-specific estimates are presented. The Joinpoint segment bars below each panel show the annual percent change (APC) for each fitted segment, and the average annual percent change (AAPC) summarizes the overall trend from 1999 to 2019. Rates are expressed per 100,000 U.S. residents and age-adjusted to the 2000 U.S. standard population. DM-nonUCD, diabetes recorded as a non-underlying cause of death.

**Supplementary Figure 4. Pre-pandemic age-adjusted mortality trends for selected underlying causes among E11-nonUCD deaths in the United States, 1999–2019.**

Annual age-adjusted mortality rates (AAMRs) are shown for deaths with the following underlying causes: (A) circulatory diseases, (B) malignant neoplasms, (C) diseases of the nervous system, (D) respiratory diseases, (E) mental and behavioral disorders, and (F) digestive diseases. Overall and sex-specific estimates are presented. The Joinpoint segment bars below each panel show the annual percent change (APC) for each fitted segment, and the average annual percent change (AAPC) summarizes the overall trend from 1999 to 2019. Rates are expressed per 100,000 U.S. residents and age-adjusted to the 2000 U.S. standard population. E11-nonUCD, type 2 diabetes recorded as a non-underlying cause of death.

**Supplementary Figure 5. Pre-pandemic age-adjusted mortality trends for selected underlying causes among E10-nonUCD deaths in the United States, 1999–2019.**
Annual age-adjusted mortality rates (AAMRs) are shown for deaths with the following underlying causes: (A) circulatory diseases, (B) malignant neoplasms, (C) diseases of the nervous system, (D) respiratory diseases, (E) mental and behavioral disorders, and (F) digestive diseases. Overall and sex-specific estimates are presented. The Joinpoint segment bars below each panel show the annual percent change (APC) for each fitted segment, and the average annual percent change (AAPC) summarizes the overall trend from 1999 to 2019. Rates are expressed per 100,000 U.S. residents and age-adjusted to the 2000 U.S. standard population. E10-nonUCD, type 1 diabetes recorded as a non-underlying cause of death.
